# Sex‐dependent role of D2‐type dopamine receptors in the dorsal hippocampus in social learning and social interactions in mice

**DOI:** 10.1111/jne.70326

**Published:** 2026-11-13

**Authors:** Noah Bass, Emily A. Underwood, Zoe K. Leach, Alexander C. Vertes, Elena Choleris

**Affiliations:** ^1^ Department of Psychology and Neuroscience Program University of Guelph Guelph Ontario Canada; ^2^ Present address: Michigan Medicine, Department of Internal Medicine, Division of Cardiology University of Michigan Ann Arbor Michigan USA

**Keywords:** dorsal hippocampus, raclopride, sex difference, social behaviors, social learning

## Abstract

Social learning is an adaptive and extremely common form of learning that remains poorly understood. Findings with systemic treatments in adult female mice implicated the dopaminergic (DA) system. A D1‐type DA receptor antagonist blocked social learning in the social transmission of food preference (STFP) test, whereas a D2‐type DA receptor antagonist reduced feeding. A subsequent investigation found that dorsal hippocampus (DH) microinfusions of the D1‐type DA receptor antagonist blocked the STFP in male and female mice; however, the role of D2‐type DA receptors in the hippocampus in social learning remains unknown. Here, we bilaterally infused the D2‐type DA receptor antagonist raclopride (at 10, 14, 18, or 20 μg/μL) or a saline solution (0.9% NaCl) control into the DH of adult male and female CD1 mice prior to the STFP test. Findings revealed a block in social learning in female but not male mice by raclopride at 18 and 20 μg/μL which could not be explained by effects of the drug on total food intake, oronasal investigation (i.e., exposure to the socially carried diet), or olfactory discrimination. Intra‐DH raclopride also sex‐dependently mediated social interactions, whereby males had a greater reduction in agonistic‐type behaviors, whereas females had a greater reduction in social investigatory‐type behaviors. This study demonstrates that D2‐type DA receptors in the DH sex‐dependently regulate social learning and social interactions, with female mice showing greater sensitivity than male mice to D2 receptor antagonism within the dose range tested, highlighting important sex differences in the regulation of social cognition and social interactions.

## Graphical abstract

This study shows that D2‐type DA receptors in the DH sex‐dependently regulate social learning whereby D2‐type DA receptor antagonism impaired social learning in female but not male mice at the range of doses tested and social interactions, highlighting important sex differences in the regulation of social cognition and social interactions.

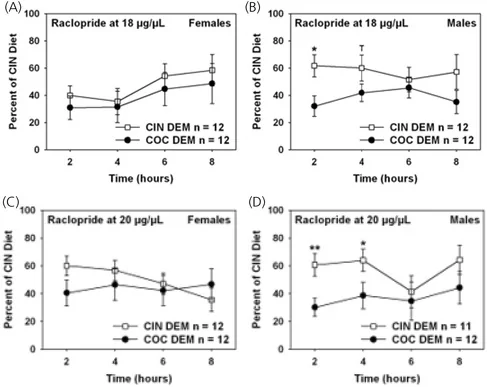

## 1 INTRODUCTION

Social learning is a prominent way of learning, especially in our own species.^1^ Yet relatively little is known of its underlying neurobiological mechanisms, with most studies on learning focusing on individual learning [rev^2^]. Social learning, defined as “learning that is influenced by observation of, or interaction with, another animal (typically a conspecific) or its products”^3^, ^4^, ^5^ allows exploiting the ‘expertise of others’.^6^ Social learning is extremely common in social species (including laboratory mice and rats) and helps limit the potential harms of trial‐and‐error individual learning.^7^ The social transmission of food preference (STFP) paradigm is a well‐established test of social learning^8^ in which the preference for a novel diet is transferred from a demonstrator (DEM) to an observer (OBS) during a social interaction.^8^ In the STFP, OBS rats and mice need to detect the flavored food odor mixed with carbon disulfide (chemical associated with digestion)^9^ found on the breath of the DEM^10^, ^11^ making oronasal investigation critical for the STFP in mice^12^ and rats.^13^

Some neurotransmitters (acetylcholine, glutamate, oxytocin/vasopressin, and opioid) and brain regions (orbitofrontal, frontal and piriform cortices, and basal forebrain) mediate social learning in the STFP [rev^14^, ^15^, ^16^]. Specifically, dopamine (DA) is important for the STFP.^17^, ^18^, ^19^ DA transporter knock‐out mice either show an opposite food preference,^19^ or no food preference,^20^ in the STFP. Furthermore, systemic administration of the D1‐type DA receptor (D1, D5) antagonist SCH 23390 blocked social learning, whereas the D2‐type DA receptor (D2, D3, and D4) antagonist raclopride reduced total food intake in female mice.^17^

The ventral tegmental area (VTA) sends direct DA projections to numerous limbic structures, including the amygdala, nucleus accumbens, and hippocampus (HPC).^21^, ^22^ The HPC is necessary for the initial encoding/acquisition of the STFP.^23^, ^24^, ^25^ Notably, dorsal hippocampal (DH) infusions of the D1‐type DA receptor antagonist SCH 23390 blocked social learning in both male and female mice without affecting total food intake, olfactory discrimination, or oronasal investigation.^18^ D2‐type DA receptors play a role in hippocampal‐dependent information processing,^26^ and D2‐type DA receptors in other brain regions (such as in the amygdala) are implicated in individually acquired food preferences.^27^ Moreover, D2‐type DA receptors are involved in the regulation of many social behaviors including social bonding,^28^ aggression,^29^ observational fear learning,^30^ and social hierarchies.^31^ However, to the best of our knowledge, the role of DH D2‐type DA receptors in social learning has not been investigated. Given the role of D2‐type DA receptors in social behavior and individually acquired food preferences, and the critical role of the DH in the STFP, it can be hypothesized that DH D2‐type DA receptors regulate the STFP.

This study investigated whether DH infusions of the D2‐type DA receptor antagonist raclopride would block social learning in the STFP. We also investigated the possible effects of raclopride on total food intake and olfactory discrimination. A full ethological analysis^32^ was conducted on the social interactions (where social learning occurs), assessing numerous behaviors including oronasal investigation. Given that our previous DA work found clear sex differences in the response to DH D1‐type DA receptor antagonists,^18^ we studied both males and females. Since estrogens and the estrous cycle regulate the STFP in mice,^15^, ^17^, ^32^ and estrogens and progesterone mediate the mesolimbic DA system,^33^ we also investigated possible effects of the estrous cycle.

## 2 METHODS AND MATERIALS

See Matta et al.^18^ and supplementary materials for full details [Tables S1, S2, Figures S1, S2, S3, and Materials and Methods].

### 2.1 Animals

Male and female 2–3‐month‐old CD‐1 mice (*Mus musculus*) were obtained from Charles Rivers (St. Constant, QC, Canada). Mice were housed in clear polyethylene cages (26 × 16 × 12 cm^3^) that were stocked with corncob bedding, plastic shelters, paper enrichment material, ad libitum food (Teklad Global 14% Protein Rodent Maintenance Diet, Harlan Teklad, WI), and tap water. The colony room was kept at 21 ± 1°C, with 40%–50% humidity, and a reversed light/dark cycle (12:12 h, lights off at 08:00). DEMs were reused (~9–14 times) for the STFP, and all OBSs and mice tested for olfactory discrimination were experimentally naïve. All experimental procedures were approved by the University of Guelph Animal Care and Use Committee and followed the guidelines of the Canadian Council on Animal Care.

### 2.2 Surgeries

For a detailed description of the surgical procedures, see Supplementary Materials (Sections: General Surgical Procedures, Ovariectomy Surgery, Castration Surgery, and Cannulation Surgery). Briefly, all DEMs were gonadectomized to ensure their hormonal status would not influence the social interactions with the OBSs. OBSs and mice tested for the olfactory discrimination task remained gonadally intact to investigate estrous cycle effects^17^, ^18^, ^32^ and were implanted with bilateral cannulae into the DH.

### 2.3 Diets

One percent ground cinnamon (CIN; McCormick Ground Cinnamon, McCormick Canada, London, Canada) or 2% ground cocoa (COC; Fry's Premium Cocoa, Cadbury, Mississauga, Canada) mixed with ground rodent chow was used. The CIN and COC diets have similar physical and metabolic characteristics and are equally palatable to CD‐1 mice.^17^, ^32^

### 2.4 Drug

The D2‐type DA receptor antagonist raclopride tartrate^34^ (Sigma‐Aldrich, Oakville, ON, Canada) was dissolved in sterile saline solution (0.9% NaCl). Ten minutes prior to testing, mice were infused with 1.0 μL (0.5 μL per hemisphere) at a flow rate of 0.2 μL per minute using a microinfusion pump (PHD 2000 injector, Harvard Apparatus, QC, Canada).

### 2.5 Experimental procedures

Social transmission of food preferences: OBSs received DH infusions of either the saline solution (0.9% NaCl) control or one of four doses of raclopride (at 10, 14, 18, or 20 μg/μL) 10‐min prior to a 30‐min social interaction with a DEM that had just consumed either the COC or the CIN diet for 1 h. OBS then immediately received an 8 h choice test between the COC and CIN diets.

Olfactory discrimination task and assessment of cannulae placement: Details in Matta et al.,^18^ and Supplementary Materials (Figure S2, Section: Olfactory Discrimination Task (ODT)).

Data handling and statistical analysis: The consumption of the COC and CIN diets was measured at 2 h‐intervals over the 8 h food choice test for each OBS, then two ratios were calculated: a preference for the CIN diet (to assess social learning within each group of treatment) and a preference for the demonstrator diet (to compare the strength of socially acquired food preferences between groups) [as theoretically expanded on in rev^49^]. Because these two measures capture complementary aspects of the STFP, we discuss the corresponding findings (Figures 1 and 2) together throughout. Total food consumed by the OBS (COC + CIN) and various behaviors performed during the social interaction (Table S1) were also analyzed. ANOVA, post hocs and planned mean comparisons were used with appropriate transformation when necessary (detailed in Supplementary Materials, Section: Data Handling and Statistical Analyses). Consistent with our previous work,^18^ overall ANOVAs were conducted for all models to reduce the risk of Type 1 errors, and only specific binary mean comparisons throughout were planned a priori rather than as exploratory post hocs, greatly reducing the total number of comparisons. Corrected post hoc multiple comparisons (e.g., Bonferroni) were not used, as such corrections are highly conservative and often increase the risk of Type 2 errors (see Supplementary Materials, Section: Data Handling, Statistical Analyses for further details and supporting references).

**FIGURE 1:**
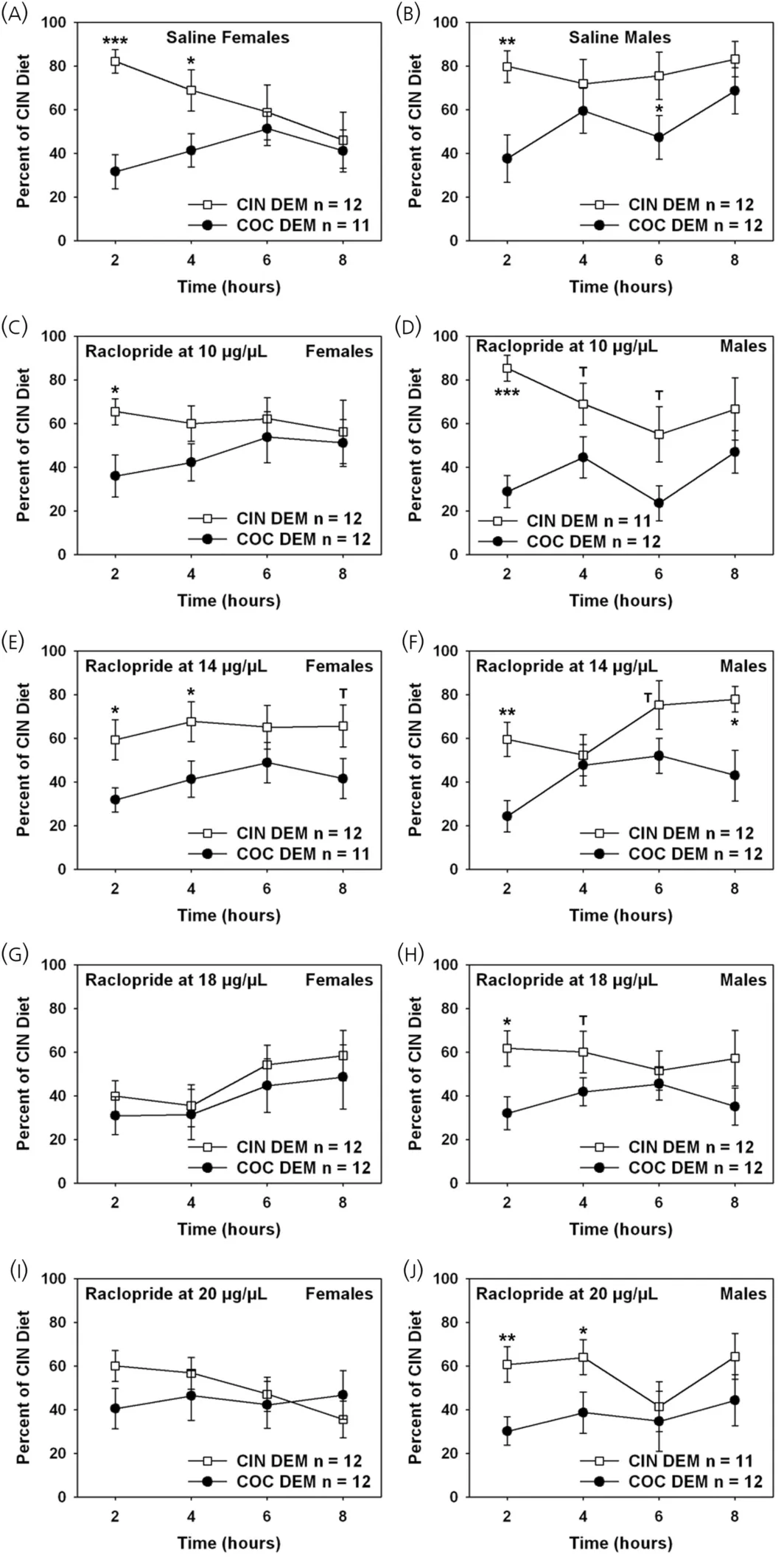
Percent of cinnamon (CIN) diet (100 × [CIN/ total amount of food consumed]) for observer (OBS) female (A, C, E, G, I) and male (B, D, F, H, J) mice that received a single DH microinfusion of either saline vehicle (A, B), or the D2‐type dopamine receptor antagonist Raclopride at 10 μg/μL (C, D), 14 μg/μL (E, F), 18 μg/μL (G, H) or 20 μg/μL (I, J) 10‐min prior to the 30‐min social interaction with a same‐sex demonstrator (DEM) that recently consumed either a 1% ground CIN (white squares) or 2% ground cocoa (COC; black circles) flavored diet. Percent of CIN diets are displayed at 2, 4, 6 and 8 h into the OBS choice test. The *n*'s represent the number of OBS mice in each group. Data are presented as mean ± SEM. *p < .05, **p < .01, ***p < .001, *T* = .05 < *p* < .1, in comparison between OBS mice that socially interacted with a DEM that consumed CIN versus OBS mice that socially interacted with a DEM that consumed COC.

**FIGURE 2:**
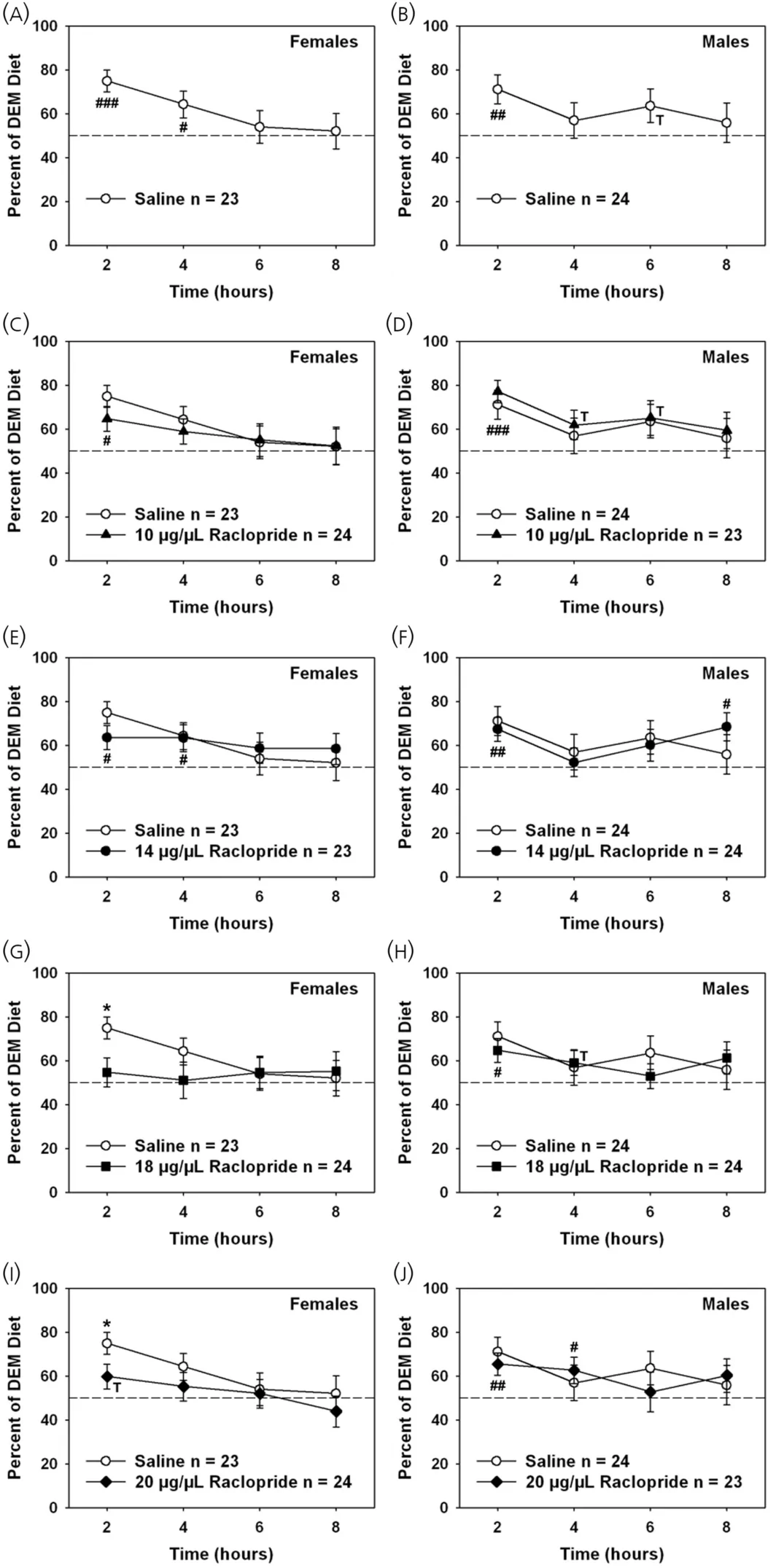
Percent of demonstrator (DEM) diet (100 × [DEM food/total amount of food consumed]) for observer (OBS) female (A, C, E, G, I) and male (B, D, F, H, J) mice that received a single DH microinfusion of either saline vehicle (white circles; A, B), or the D2‐type dopamine receptor antagonist raclopride at 10 μg/μL (black triangles; C, D), 14 μg/μL (black circles; E, F), 18 μg/μL (black squares; G, H) or 20 μg/μL (black diamonds; I, J) 10‐min prior to the 30‐min social interaction with a same‐sex DEM that recently consumed either a 1% ground cinnamon (CIN) or 2% ground cocoa (COC) flavored diet. Percent of DEM diets are displayed at 2, 4, 6 and 8 h into the OBS choice test. The *n*'s represent the number of OBS mice in each group. Data are presented as mean **±** SEM. *p < .05, in comparison between OBS mice that received an infusion of saline vehicle versus OBS mice that received an infusion of raclopride. ^#^ *p* < .05, ^##^ *p* < .01, ^###^ *p* < .001, *T* = .05 < *p* < .1, in comparison to 50% (chance).

## 3 RESULTS

### 3.1 Social transmission of food preference: Intrahippocampal raclopride blocks social learning and reduces the strength of the socially acquired food preference without influencing total food intake in female but not male mice

DH raclopride blocked social learning in females infused with 18 and 20 μg/μL (Figure 1). OBS infused with raclopride at 18 and 20 μg/μL had a weaker DEM food preference than saline infused OBS (Figure 2), and there were no overall treatment effects of DH raclopride on total food intake for either sex (Figure S6). The estrous cycle may have interacted with intrahippocampal raclopride to influence female social learning.

The RM ANOVA conducted on the preference of the CIN diet of OBS mice (Figure 1) revealed a significant time × DEM food interaction (*F*(3, 396) = 4.35, *p* = .005), significant main effects of DEM food (*F*(1, 132) = 25.93, *p* < .001), treatment (*F*(4, 132) = 2.36, *p* = .05), and a trend towards a main effect of sex (*F*(1, 132) = 3.33, *p* = .070) for the full 8 h.

Independent samples *t*‐tests comparing the percent of CIN diets of OBSs that interacted with a CIN versus COC DEM revealed a social learning impairment for females infused with raclopride at 18 and 20 μg/μL. Specifically, at 2 h there was a significant difference between the percent of CIN diets of OBS mice that interacted with a CIN DEM versus COC DEM for females (*t*(19) = 5.01, *p* < .001) and males (*t*(18) = 3.15, *p* = .006) infused with saline, raclopride at 10 μg/μL (females: *t*(21) = 2.43, *p* = .024; males: *t*(17) = 5.51, *p* < .001), and raclopride at 14 μg/μL (females: *t*(19) = 2.59, *p* = .018; males: *t*(21) = 3.38, *p* = .003). The CIN preference was significantly different in CIN versus COC demonstrated OBS only in males infused with raclopride at 18 μg/μL (*t*(21) = 2.62, *p* = .016) and 20 μg/μL (*t*(21) = 3.02, *p* = .006). At 4 h the CIN preference of the COC and CIN demonstrated OBS was significantly different for females infused with saline (*t*(20) = 2.42, *p* = .025) and raclopride at 14 μg/μL (*t*(19) = 2.26, *p* = .036), and males infused with raclopride at 20 μg/μL (*t*(21) = 2.26, *p* = .035), while males infused with raclopride at 10 μg/μL (*t*(19) = 1.94, *p* = .067) and 18 μg/μL (*t*(22) = 1.79, *p* = .086) showed a trend towards significance. At 6 h for males infused with saline (*t*(19) = 2.14, *p* = .045), while males infused with raclopride at 10 μg/μL (*t*(15) = 1.99, *p* = .065) and 14 μg/μL (*t*(16) = 1.79, *p* = .092) still trended towards significance. At 8 h for males infused with raclopride at 14 μg/μL (*t*(18) = 2.61, *p* = .018), while there was only a trend towards significance for females infused with raclopride at 14 μg/μL (*t*(14) = 1.84, *p* = .088). Importantly, there was no significant difference between the percent of CIN diets of OBSs that interacted with a CIN versus COC DEM at any time points during the choice test for females infused with raclopride at 18 and 20 μg/μL, proving that intrahippocampal raclopride blocked social learning in females at these doses.

The RM ANOVA conducted on the preference for the DEM diet by OBS mice revealed a significant main effect of time (*F*(3, 429) = 4.30, *p* = .005). RM ANOVAs further revealed a significant main effect of time for both males (*F*(3, 213) = 2.89, *p* = .036) and females (*F*(3, 216) = 2.70, *p* = .047). Notably, independent samples *t*‐tests conducted on the preference of the DEM diet in control OBS infused with saline versus OBS infused with raclopride revealed that at 2 h female OBS infused with raclopride at 18 μg/μL (*t*(40) = 2.35, *p* = .024) and 20 μg/μL (*t*(41) = 2.05, *p* = .047) showed a significantly lower preference for the DEM diet than saline infused females. One‐sample *t*‐tests comparing the preference of the DEM diet to 50% (chance) further showed that at 2 h females (*t*(20) = 4.86, *p* < .001) and males (*t*(19) = 3.03, *p* = .007) infused with saline, females (*t*(22) = 2.49, *p* = .021) and males (*t*(18) = 5.08, *p* < .001) infused with raclopride at 10 μg/μL, females (*t*(20) = 2.59, *p* = .017) and males (*t*(22) = 3.19, *p* = .004) infused with raclopride at 14 μg/μL, and males infused with raclopride at 18 μg/μL (*t*(22) = 2.64, *p* = .015) and 20 μg/μL (*t*(22) = 3.09, *p* = .005) had a significantly higher preference for the DEM diet than 50% (chance), whereas females infused with raclopride at 20 μg/μL did not (*t*(21) = 1.75, *p* = .095). At 4 h, females infused with saline (*t*(21) = 2.52, *p* = .02) and raclopride at 14 μg/μL (*t*(20) = 2.32, *p* = .031), and males infused with raclopride at 20 μg/μL [*t*(22) = 2.32, *p* = .03] showed a significantly higher preference for the DEM diet than 50%, whereas males infused with raclopride at 10 μg/μL (*t*(20) = 1.89, *p* = .073) and 18 μg/μL (*t*(23) = 1.84, *p* = .08) were only trending towards significance. At 6 h, males infused with saline (*t*(20) = 1.94, *p* = .066) and raclopride at 10 μg/μL (*t*(16) = 1.88, *p* = .079) showed a trend towards preferring the DEM diet above 50%. At 8 h, only males infused with raclopride at 14 μg/μL (*t*(19) = 2.65, *p* = .016) preferred the DEM diet above 50%. Notably, preference for the DEM diet of females infused with raclopride at 18 and 20 μg/μL were not significantly above 50% (chance) at any time points during the choice test. Overall, female OBS mice infused with raclopride at 18 and 20 μg/μL had a lower preference for the DEM diet than saline infused control mice and were not significantly different than chance levels (50%), which demonstrates that the STFP was blocked in females at these doses.

Food consumption was not affected by DH raclopride. See details in Supplementary Materials (Figure S6, Section: Total Food Intake). Hence, the social learning impairment by DH raclopride found in this study is not secondary to changes in feeding.

### 3.2 Social transmission of food preference study: The phase of the estrous cycle affects how raclopride impacts social learning

The RM ANOVA conducted on the preference for the CIN diets of females OBS revealed a significant main effect of the estrous phase (*F*(2, 50) = 3.82, *p* = .029) and a significant time × estrous phase × DEM food interaction (*F*(6, 150) = 2.73, *p* = .015). Separate ANOVAs showed a significant estrous phase × treatment interaction at 2 h (*F*(8, 80) = 2.34, *p* = .026), and a significant estrous phase × DEM food interaction at 6 h (*F*(2, 80) = 3.42, *p* = .038). Hence, the estrous phase may have affected the female percent of CIN diets for certain time points of the choice test, as a function of their treatment group and the food their DEM ate. However, mean comparisons for each treatment group broken down by estrous phase did not yield significances, likely due to the small number of females for each phase, per treatment group (details in Supplementary Materials, Table S3). A similar trend towards an estrous phase interaction with treatment was also found for total food intake (Supplementary Materials “Phase of the Estrous Cycle” section).

### 3.3 Social transmission of food preference study: The effects of intrahippocampal raclopride on behavior during the social interactions

For detailed results see Supplementary Materials (Section: Results). Notably, oronasal investigation was not affected by intrahippocampal raclopride (Figures S9C and S9D), demonstrating that the social learning blockade by raclopride found in females was not due to changes in the time exposed to the food odor carried by the DEM. Additionally, DH raclopride also sex‐dependently mediated social interactions, whereby males had a greater reduction in agonistic‐type behaviors, whereas females had a greater reduction in social investigatory‐type behaviors (summarized in Table S5). This pattern paralleled baseline sex differences observed in saline‐treated mice, whereby males engaged in more agonistic behaviors (e.g., boxing, open aggression, and dominant behavior) than females, while females engaged in more social investigatory behaviors (e.g., body and anogenital investigation, following the DEM) than males (Tables S4 and S5). Therefore, DH raclopride reduced each sex's predominant social behavior to a greater extent, consistent with a sex‐dependent modulation of social interactions by D2‐type DA receptors. Raclopride reduced overall activity in female but not male mice, and in both sex's it reduced active social behaviors (Figure S7) such as agonistic behaviors delivered (e.g., following the DEM/dominant behavior) paralleled by an increase in non‐social inactive behavior (e.g., solitary inactivity). In males and females, raclopride reduced social investigatory behaviors (e.g., body and anogenital investigation), dominance score (e.g., subordination towards DEM), and increased non‐social behaviors (solitary inactivity; Figures S8‐S12). Overall, because DH raclopride's reduced social behavior in both sexes, this effect appears unlikely to directly explain the sex difference in its effects on social learning.

### 3.4 Olfactory discrimination study: DH raclopride did not affect the discrimination between the CIN and the COC diets by female mice

Detailed results are in Supplementary Materials (Section: Results). Briefly, the RM ANOVA conducted on the investigation ratio of female mice showed no effects of raclopride at 18 and 20 μg/μL, the two doses that blocked the STFP (Figure S14). Thus, the social learning impairment was not secondary to impaired olfactory discrimination of the COC and CIN diets.

## 4 DISCUSSION

This study found an intriguing sex difference in the regulation of social learning by D2‐type DA receptors in the DH such that at the doses tested, raclopride impaired it in female but not male mice, without affecting oronasal investigation (Figure 1), feeding behavior (Figure 2), or olfactory discrimination (Figure S14). As the CIN and DEM preference analyses reflect complementary aspects of the STFP, we discuss the corresponding findings (Figures 1 and 2) together. While oronasal investigation was not affected, effects on other aspects of social interactions may explain the findings. For example, it is possible that reduced social investigation in females may reflect reduced interest in the DEMs, including the food‐related information they carry. However, DH raclopride also affected social behavior in males, including significant reductions in social investigation, yet male social learning was not impaired at any dose (Table S5). In other words, similar effects on social behavior in both sexes did not lead to the same effects on social learning, suggesting that it is unlikely that the effects on female social interactions directly explain the block in social learning. Rather, it is more likely that the sex difference in the effects on social learning is due to a different modulation of learning mechanisms, consistent with the differential dose sensitivity between the sexes discussed above.

DH raclopride also reduced dominance score and total activity, raising the possibility that these effects contributed to the social learning impairment. However, this seems unlikely since dominance scores were reduced at all doses of raclopride in both sexes, whereas social learning was only impaired at the highest doses and only in females (Tables S4 and S5). Similarly, female total activity was reduced comparably across all doses tested (Figure S7), including doses that did not impair social learning. Together, these findings suggest that the social learning impairment observed here is not directly explained by reductions in dominance or total activity, consistent with a more specific effect of DH D2‐type DA receptor antagonism on social learning related processes. As with social investigation (as discussed above), dominance and total activity were each affected across a broader range of doses and in both sexes than social learning was. The mismatch in dose and sex specificity makes it unlikely that any single behavioral effect of DH raclopride can directly account for the social learning impairments observed.

The effects of DH raclopride on socially acquired food preferences found here are consistent with those of previous work reporting a role for other mesocorticolimbic brain regions in individually acquired food preferences. Raclopride infused into the amygdala^27^ or medial prefrontal cortex^35^ impairs the acquisition of a fructose‐conditioned flavor preference, while infusions into the NAc shell^36^ or medial orbital frontal cortex^36^ regulate its expression in rats. Hippocampal D2‐type DA receptors have also been implicated in non‐social types of learning, including spatial learning,^37^ expression of conditioned place preference,^38^ and state‐dependent learning.^39^ Hence, our results extend those findings to socially acquired food preferences and add the DH as another brain region of action through D2‐type DA receptors.

The social learning impairment found in this study may have been due to effects on learning and memory processes. The VTA‐to‐hippocampal dopaminergic loop is involved in regulating novel information signaling associated with goal directed behavior.^40^ It is possible that when female OBS in the STFP encountered a behaviorally salient stimulus (the novel food odor on the breath of the DEM), mild stimulation of the VTA inputs to the hippocampus ensues.^40^, ^41^ This signal may result in (mostly) low frequency tonic firing from the VTA to the hippocampus leading to constant (but low levels) extracellular DA, which primarily (if not exclusively) activates high affinity D2‐type DA receptors.^42^, ^43^, ^44^ Thus, socially learning a food preference for female mice may depend on tonic firing of DA neurons, where the low levels of extracellular DA in the DH must activate D2‐type DA receptors (which processes social novelty) and contribute to hippocampus dependent engram formation and/or stabilization.^40^, ^42^

Interestingly, the DH results found here in female mice are different than those of systemic treatment^17^ which reduced oronasal investigation and food intake without affecting social learning. Instead, the results of this study are in line with the DH effects of D1‐type DA receptor antagonist SCH 23390 which blocked social learning without affecting oronasal investigation or feeding behavior in either sex.^18^ Thus, the food intake results obtained with systemic treatment^17^ are mediated by a brain region other than the DH, possibly the nucleus accumbens where DA is known to affect feeding.^45^ Instead, the block of female social learning in this study, and not with systemic raclopride,^17^ may be due to an interaction of treatment with the estrous cycle and gonadal hormones, discussed below.

Only female social learning was impaired at the two highest doses of raclopride, whereas males did not respond to any of the doses used here. It remains possible that social learning in male mice could have been affected by raclopride at doses higher than 20 μg/μL. Thus, rather than directly demonstrating an exclusive role for D2‐type DA receptors in female social learning, our findings may reflect a sex difference in dose–response sensitivity, with females showing greater sensitivity than males to DH D2‐type DA receptor antagonism within the range tested. We did not test doses above 20 μg/μL because we were concerned that higher doses of raclopride could produce a range of non‐specific effects on general activity or behavioral state, which could complicate the interpretation of changes in social learning.

The sex difference in the effects of raclopride on social learning is in the opposite direction to that found in our previous DH study showing that male mice were more sensitive to a D1‐type antagonist in the DH than females. Blocking D1‐type DA receptors in the HPC impaired social learning with different dose response in the two sexes; the lowest and two highest doses a D1‐type antagonist blocked it in males, whereas only the highest dose was effective in females.^18^ Thus, our previous^18^ and current DH results, collectively conclude that male social learning is mediated by DH D1‐type receptors, whereas in females social learning DH D1‐type receptors are less involved, and instead, D2‐type DA receptors are also implicated.

The finding that DH raclopride affected social learning in female but not male mice, together with the effects of the estrous cycle reported above, is consistent with previous findings showing that estrogens modulate the STFP^15^, ^32^ and the dopaminergic system,^50^ raising the possibility of an involvement of gonadal hormones. Either testicular hormones protect from the impairing effects of DH raclopride, or ovarian hormones enhance those effects (or a combination of the two). Indeed, our lab finds clear effects of estrogens and their receptors on the STFP in mice.^15^, ^32^ Additionally, the estrous cycle affects both the STFP^17^ and mesolimbic DA activity.^33^ Accordingly, the present study found that the estrous cycle significantly affected the CIN preference, both as a main effect, and in interactions with time, treatment, and DEM food (see Section 3). The estrous cycle also interacted with time to affect the DEM preference, and showed a trend towards an interaction with treatment on total food intake (further detailed in Supplementary Materials). Further mean comparisons for each treatment group by estrous phase did not yield any significances, likely because of the small number of females in each estrous phase, per treatment group (Table S3). We opted not to collapse the proestrus and estrus phases of the cycle into a single category because these phases are characterized by markedly different hormonal profiles. Proestrus involves high circulating estrogens and progesterone whereas both hormones decline during estrus. Furthermore, our laboratory's previous work has shown that these phases can be associated with distinct behavioral effects on social learning [e.g.,^15^, ^17^]. Keeping these phases separate here allows for the possibility of detecting similar phase‐specific effects which could be precluded by collapsing the two groups. We also note that combining these groups would not have meaningfully improved statistical power given the low sample sizes of each phase overall (see Table S3). Given that male social learning was unaffected by intra‐hippocampal raclopride, it is possible that testosterone has neuroprotective effects [see^46^]. There is evidence from studies conducted on Alzheimer's disease, and mild cognitive impairment (e.g., dementia) to support this hypothesis, however, testosterone may not always protect against neurotoxicity of the DA system.^47^ Further research assessing the involvement of gonadal hormones in the dopaminergic mediation of social learning in both sexes is needed.

In summary, this study found that antagonizing DH D2‐type DA receptors blocked the STFP in female mice at doses that did not impair social learning in males, indicating that social learning is more sensitive to DH D2‐type DA receptor antagonism within the range tested in females. This impairment could not be explained by any secondary effects of drug treatment on feeding behavior, oronasal investigation, or olfactory discrimination. Further studies examining possible interactions with gonadal hormones would be valuable.

## AUTHOR CONTRIBUTIONS

**Noah Bass:** Conceptualization; investigation; funding acquisition; writing – original draft; methodology; validation; visualization; writing – review and editing; formal analysis; data curation. **Emily A. Underwood:** Methodology. **Zoe K. Leach:** Methodology. **Alexander C. Vertes:** Methodology. **Elena Choleris:** Funding acquisition; methodology; writing – review and editing; formal analysis; project administration; supervision; data curation; writing – original draft; validation; conceptualization.

## FUNDING INFORMATION

This work was supported by the Natural Sciences and Engineering Research Council of Canada [RGPIN‐2018‐04699].

## CONFLICT OF INTEREST STATEMENT

The authors declare no conflicts of interest.

## Supporting information


**Table S1:** Single behaviors scored for the 30‐min social interactions in the STFP [from^2^, ^42^].
**Table S2:** Grouped behaviors scored for the 30‐min social interactions in the STFP [from^2^].
**Figure S1:** Mouse brain cannula placements for observer mice in the social transmission of food preference paradigm.
**Figure S2:** Mouse brain cannula placement for mice in the olfactory discrimination task.
**Figure S3:** Photomicrograph (at 3.2× magnification) of a coronal slice of the mouse dorsal hippocampus following an infusion of 1% Chicago blue dye in phosphate buffered saline. Arrow signifies the site of the microinjection.
**Figure S4:** Timeline for the intra‐hippocampal Raclopride social transmission of food preferences (STFP) experiment. “DEM” stands for demonstrator mouse; “OBS” stands for observer mouse.
**Figure S5:** Timeline for the intra‐hippocampal Raclopride olfactory discrimination task (ODT). “Hab” stands for habituation phase.
**Table S3:** Sample sizes (*n*'s) for each estrous phase, per treatment group, for the social transmission of food preferences (STFP) DH Raclopride experiments.
**Figure S6:** Total food intake (1% ground cinnamon [CIN] + 2% ground cocoa [COC]) for observer (OBS) female (A) and male (B) mice that received a single DH microinfusion of either saline vehicle (white circles; *n* = 23 females and *n* = 24 males), or the D2‐type dopamine receptor antagonist Raclopride at 10 μg/μL (black triangles; *n* = 24 females and *n* = 23 males), 14 μg/μL (black circles; *n* = 23 females and *n* = 24 males), 18 μg/μL (black squares; *n* = 24 females and *n* = 24 males) or 20 μg/μL (black diamonds; *n* = 24 females and *n* = 23 males) 10‐min prior to the 30‐min social interaction with a same‐sex demonstrator (DEM) mouse that recently consumed either a CIN or COC flavored diet. Total food intakes are displayed at 2, 4, 6 and 8 h into the OBS choice test. There were no significant effects of DH raclopride on total food intakes for either sex. Data are presented as mean **±** SEM.
**Figure S7:** Active behaviors (in sec) for observer (OBS) female [A, C] and male [B, D] mice that received a single DH microinfusion of either saline vehicle (*n* = 23 females and *n* = 24 males), or the D2‐type dopamine receptor antagonist raclopride at 10 μg/μL (*n* = 24 females and *n* = 23 males), 14 μg/μL (*n* = 23 females and *n* = 24 males), 18 μg/μL (*n* = 24 females and *n* = 24 males) or 20 μg/μL (*n* = 24 females and *n* = 23 males). Infusions were administered 10 min prior to a 30 min social interaction where OBS mice had the opportunity to acquire a food preference from a same‐sex demonstrator (DEM) mouse that recently consumed either a 1% ground cinnamon (CIN) or 2% ground cocoa (COC) flavored diet. DH raclopride effects on female [A] and male [B] total activity, and female [C] and male [D] total social behavior are shown. There were no significant effects of DH raclopride on male total activity [B]. Data are presented as mean + SEM. *p < .05, **p < .01, ***p < .001, in comparison to saline infused control animals.
**Figure S8:** Agonistic behaviors for observer (OBS) male mice that received a single DH microinfusion of either saline vehicle (*n* = 24), or the D2‐type dopamine receptor antagonist raclopride at 10 μg/μL (*n* = 23), 14 μg/μL (*n* = 24), 18 μg/μL (*n* = 24) or 20 μg/μL (*n* = 23). Infusions were administered 10 min prior to a 30 min social interaction where OBS mice had the opportunity to acquire a food preference from a same‐sex demonstrator (DEM) mouse that recently consumed either a 1% ground cinnamon (CIN) or 2% ground cocoa (COC) flavored diet. DH raclopride effects on male total agonistic behaviors [A], dominance score [B], dominant behavior [C], boxing [D], submissive behavior [E], agonistic behavior received [F], and agonistic behavior delivered [G] are shown. Data are presented as mean + SEM. *p < .05, **p < .01, ***p < .001, *T* = .05 < *p* < .1, in comparison to saline infused control animals.
**Figure S9:** Social investigatory behaviors (in sec) for observer (OBS) female [A, C, E, G, I] and male [B, D, F, H, J] mice that received a single DH microinfusion of either saline vehicle (*n* = 23 females and *n* = 24 males), or the D2‐type dopamine receptor antagonist raclopride at 10 μg/μL (*n* = 24 females and *n* = 23 males), 14 μg/μL (*n* = 23 females and *n* = 24 males), 18 μg/μL (*n* = 24 females and *n* = 24 males) or 20 μg/μL (*n* = 24 females and *n* = 23 males). Infusions were administered 10 min prior to a 30‐min social interaction where OBS mice had the opportunity to acquire a food preference from a same‐sex demonstrator (DEM) mouse that recently consumed either a 1% ground cinnamon (CIN) or 2% ground cocoa (COC) flavored diet. DH raclopride effects on female and male social investigation [A, B], oronasal investigation [C, D], body investigation [E, F], anogenital investigation [G, H], and following the DEM [I, J] are shown. There were no significant effects of DH raclopride on either female [C] or male [D] oronasal investigation, or following the DEM for males [J]. Data are presented as mean + SEM. *p < .05, **p < .01, ***p < .001, *T* = .05 < *p* < .1, in comparison to saline infused control animals.
**Figure S10:** Agonistic behaviors (in sec) for observer (OBS) female mice that received a single DH microinfusion of either saline vehicle (*n* = 23), or the D2‐type dopamine receptor antagonist raclopride at 10 μg/μL (*n* = 24), 14 μg/μL (*n* = 23), 18 μg/μL (*n* = 24) or 20 μg/μL (*n* = 24). Infusions were administered 10 min prior to a 30‐min social interaction where OBS mice had the opportunity to acquire a food preference from a same‐sex demonstrator (DEM) mouse that recently consumed either a 1% ground cinnamon or 2% ground cocoa flavored diet. DH raclopride effects on female agonistic behavior delivered [A], dominant behavior [B], and dominance score [C] are shown. Data are presented as mean + SEM. *p < .05, **p < .01, ***p < .001, *T* = .05 < *p* < .1, in comparison to saline infused control animals.
**Figure S11:** Non‐social behaviors (in sec) for observer (OBS) female [A] and male [B] mice that received a single DH microinfusion of either saline vehicle (*n* = 23 females and *n* = 24 males), or the D2‐type dopamine receptor antagonist raclopride at 10 μg/μL (*n* = 24 females and *n* = 23 males), 14 μg/μL (*n* = 23 females and *n* = 24 males), 18 μg/μL (*n* = 24 females and *n* = 24 males) or 20 μg/μL (*n* = 24 females and *n* = 23 males). Infusions were administered 10 min prior to a 30‐min social interaction where OBS mice had the opportunity to acquire a food preference from a same‐sex demonstrator (DEM) mouse that recently consumed either a 1% ground cinnamon (CIN) or 2% ground cocoa (COC) flavored diet. Data are presented as mean + SEM. *p < .05, **p < .01, ***p < .001, in comparison to saline infused control animals.
**Figure S12:** Non‐social non‐locomotor behaviors (in sec) for observer (OBS) female [A, C, E] and male [B, D, F] mice that received a single DH microinfusion of either saline vehicle (*n* = 23 females and *n* = 24 males), or the D2‐type dopamine receptor antagonist raclopride at 10 μg/μL (*n* = 24 females and *n* = 23 males), 14 μg/μL (*n* = 23 females and *n* = 24 males), 18 μg/μL (*n* = 24 females and *n* = 24 males) or 20 μg/μL (*n* = 24 females and *n* = 23 males). Infusions were administered 10 min prior to a 30 min social interaction where OBS mice had the opportunity to acquire a food preference from a same‐sex demonstrator (DEM) mouse that recently consumed either a 1% ground cinnamon (CIN) or 2% ground cocoa (COC) flavored diet. DH raclopride effects on female and male non‐social non‐locomotor behaviors [A, B], solitary inactivity [C, D], and self‐grooming [E, F] are shown. There were no significant effects of DH raclopride on female self‐grooming [E]. Data are presented as mean + SEM. *p < .05, **p < .01, ***p < .001, in comparison to saline infused control animals.
**Table S4:** Summarized DH raclopride effects on grouped behaviors during the 30 min social interactions for the STFP experiment.
**Table S5:** Summarized DH raclopride effects on single behaviors during the 30 min social interactions for the STFP experiment.
**Figure S13:** Baseline sex differences for observer (OBS) mice that received a single DH microinfusion of saline vehicle (*n* = 23 females [black bars] and *n* = 24 males [white bars]). Infusions were administered 10 min prior to a 30 min social interaction where OBS mice had the opportunity to acquire a food preference from a same‐sex demonstrator (DEM) mouse that recently consumed either a 1% ground cinnamon (CIN) or 2% ground cocoa (COC) flavored diet. Sex differences are shown for various grouped and single behaviors for duration [A] and frequency [B] data. “Ago Rec” stands for agonistic behavior received; “Tot Ago” stands for total agonistic behaviors; “Dom Score” stands for dominance score; “Soc Inves” stands for social investigation; “Dominant Behav” stands for dominant behavior; “Open Aggr” stands for open aggression; “Submissive” stands for submissive behavior; “Body Inves” stands for body investigation; “Anogenital Inves” stands for anogenital investigation; “Ago Del” stands for agonistic behavior delivered; “Follow” stands for following the DEM; “Attacks Del” stands for attacks delivered; “Sol Inactivity” stands for solitary inactivity. Data are presented as mean + SEM. *p < .05, **p < .01, ***p < .001, *T* = .05 < *p* < .1, saline infused females versus saline infused males.
**Figure S14:** DH raclopride olfactory discrimination task (ODT). Female mice received a single DH microinfusion of either saline vehicle (*n* = 12), or the dopamine (DA) D2‐type receptor antagonist raclopride at 18 μg/μL (*n* = 11) or 20 μg/μL (*n* = 12). Infusions were administered 10 min prior to four habituation phases and one test phase. During the habituation phases (black bars) mice were exposed to two jars (covered with wire mesh to prevent consumption) containing the same food odors [i.e., two jars containing 1% ground cinnamon (CIN), or two jars containing 2% ground cocoa (COC)]. For the test session (gray bars) one of the formerly presented familiar diets was exchanged for a novel diet (i.e., mice were exposed to both CIN and COC). Each phase was 5 min in duration, and was administered consecutively in the home cage. For graph A: DH raclopride effects on female percent of novel diet investigation (NDI) scores ([time investigating novel diet / total time investigating both diets] × 100); data are presented as mean + SEM; *p < .05, ***p < .001, averaged NDI scores of the four habituation phases versus test NDI scores. For graph B: DH raclopride effects on female total investigation [time investigating familiar diet + time investigating novel diet]; data are presented as mean ± SEM; *p < .05, **p < .01, *T* = .05 < *p* < .1, in comparison to saline infused control animals.
**Table S6:** Sample sizes (*n*'s) for each estrous phase, per treatment group, for the olfactory discrimination task (ODT) DH raclopride experiments.

## Data Availability

The data that support the findings of this study are available from the corresponding author upon reasonable request.
