# Supplementary material for: Sex‐dependent role of D2‐type dopamine receptors in the dorsal hippocampus in social learning and social interactions in mice
Source: J Neuroendocrinol. 2026 Nov 13;38(11):e70326. doi: 10.1111/jne.70326 (PMC13762377; doi:10.1111/jne.70326)
Supplement: Supplementary file 1 [file JNE-38-e70326-s001.docx]

**SUPPLEMENTAL INFORMATION**

This document contains supplementary information regarding the methods, experimental procedures, data handling, statistical analyses, results, and a complete discussion on the effects of intra-dorsal hippocampal (DH) raclopride on social/non-social behaviors during the social interactions of the social transmission of food preference (STFP) paradigm.

**Table S1.** Single behaviors scored for the 30-min social interactions in the STFP [from 2, 43].

| **Social Behaviors** | |
| --- | --- |
| **Following the DEM** | OBS actively follows, or pursues and chases the DEM; reciprocal to avoid. |
| **Dominant Behavior** | The OBS is in control; includes pinning of DEM, aggressive grooming, crawling over or on top, and mounting attempt. |
| **Attacks Delivered** | Physical attacks, including dorsal/ventral bites. Only frequency of attacks was measured. |
| **Boxing** | Physical attacks which include box/wrestle, offensive and defensive postures, lateral sideways threats and tail rattle. |
| **Open Aggression** | Physical attacks with a locked fight including tumbling, kick-away and counterattack where the attacker cannot be identified. |
| **Avoidance of DEM** | OBS withdraws and runs away from DEM while DEM is following. |
| **Submissive Behavior** | DEM is in control; includes crawl under, supine posture (ventral side exposed), prolonged crouch, and any other behavior in which the DEM is dominant (e.g., DEM pins, aggressively grooms, etc, OBS). |
| **Attacks Received** | Physical attacks including bites to dorsal/ventral regions. Only frequency of attacks was measured. |
| **Defensive Upright Posturing** | Species-typical defensive behavior; upright with the head tucked and the arms ready to push away. |
| **Social Inactivity** | Includes sit/lie/sleep together. |
| **Oronasal Investigation** | Active sniffing of DEM’s oronasal area. |
| **Body Investigation** | Active sniffing of DEM’s body. |
| **Anogenital investigation** | Active sniffing of DEM's anogenital region. |
| **Stretched Approach** | Risk assessment behavior; back feet do not move and front feet approach DEM. Only frequency of stretched approaches was measured. |
| **Approaching and/or Attending to the DEM** | Often from across the cage; OBS's attention is focused on DEM, head tilted toward DEM and movements toward DEM; this becomes 'Follow Opponent' once along the tail or sniff. |
| **Non-social Behaviors** | |
| **Horizontal Exploration** | Movement around the cage; includes active sniffing of air and ground. |
| **Vertical Exploration** | Movement to investigate upwards, both front feet off the ground; includes sniffing, wall leans and lid chews (less than 3). |
| **Digging** | Rapid stereotypical movement of forepaws in the bedding. |
| **Abnormal Stereotypies** | “Strange” behaviors, including spinturns, repeated jumps/lid chews/head shakes (more than 3). |
| **Solitary Inactivity** | No movement; includes sit, lie down and sleep. |
| **Self-Grooming** | Rapid movement of forepaws over facial area and along body. |

**Table S2.** Grouped behaviors scored for the 30-min social interactions in the STFP [from 2].

| **Total Activity** | All behaviors involving activity, both social and non-social. Excluded from this group are Inactive Alone, Inactive Together, and Self-Groom. |
| --- | --- |
| **Total Social Behavior** | This composite behavior does not indicate whether the social interactions are affiliative or agonistic. It includes: Follow DEM, Dominant Behavior, Attack Delivered, Boxing, Open Aggression, Avoid DEM, Submissive Behavior, Attack Received, Defensive Upright Posture, Inactive Together, Oronasal Investigation, Body Investigation, Anogenital investigation, Stretched Approach, and Attend to/approach DEM. |
| **Agonistic Behavior Delivered** | Follow Demonstrator, Dominant Behavior, and Attack Delivered. |
| **Agonistic Behavior Received** | Avoid DEM, Submissive Behavior, Attack Received, and Defensive Upright Posture. |
| **Total Agonistic Behaviors** | This composite behavior does not indicate the direction of the agonistic behavior (i.e., whether agonistic behavior is directed toward OBS or toward DEM). It includes Agonistic Behavior Delivered and Received plus Open Aggression, and Boxing. |
| **Dominance Score** | Total agonistic behavior delivered minus total agonistic behavior received. A negative score indicates that the OBS was the submissive animal in the pair, while a positive score signifies that the OBS was the dominant animal. |
| **Social Investigation** | Oronasal Investigation, Body Investigation, Anogenital investigation, Stretched Approach, and Attend to/approach DEM. |
| **Non-social Behaviors** | Horizontal Exploration, Vertical Exploration, Dig, Stereotypies, Inactive Alone, and Self-Groom. |
| **Non-social Locomotor Behaviors** | Horizontal Exploration, Vertical Exploration, and Dig. |
| **Non-social Non-locomotor Behaviors** | Inactive and Self-Groom. |

*Mouse Brain Cannula Placements for the STFP and ODT DH Raclopride Experiments*

For the STFP (Figure S1) and ODT (Figure S2) DH raclopride experiments, most injectors were found in the anterior dorsal hippocampus (between -1.70mm and -1.82mm), however some were more posterior (-2.46mm) though still within the DH. The locations of the cannula placements did not appear to differ between groups (for either sex).

**Figure S1.** Mouse brain cannula placements for observer mice in the social transmission of food preference paradigm.

**Figure S2.** Mouse brain cannula placement for mice in the olfactory discrimination task.

**Figure S3.** Photomicrograph (at 3.2x magnification) of a coronal slice of the mouse dorsal hippocampus following an infusion of 1% Chicago blue dye in phosphate buffered saline. Arrow signifies the site of the microinjection.

**MATERIALS AND METHODS**

**Animals.** Upon arrival to the colony room, mice were triple housed (with the same sex). All mice had at least one week to adjust to the colony room before starting any experiments. Ten days post surgery and 3 days before testing mice were double housed with a same-sex animal for the STFP (separated by a perforated partition described below) or singly housed for the olfactory discrimination task (ODT) in the same polyethylene cages.

**General Surgical Procedures.** All DEMs in the STFP were gonadectomized to ensure that any effects on the OBSs social learning in the STFP was due to the drug manipulations, and not to the hormonal status of the DEM during the social interactions. OBSs and ODT mice had bilateral guide cannulas implanted, but were left gonadally intact to investigate any interactions between gonadal hormones and the effects of raclopride on the STFP [see 1, 2, 3] and diet odor discrimination. DEM mice were reused (approximately 9 to 14 times) while OBSs in the STFP, and mice tested in the ODT were always experimentally naïve.

All surgeries involved mice first receiving an injection of carprofen at 50mg/kg (analgesic/anti-inflammatory agent, subcutaneous [s.c.] injection; Rimadyl, Pfizer Canada Inc, Kirkland, QC, Canada). Thirty minutes later, mice were anaesthetized with isoflurane (Benson Medical Industries, Markham, ON), and were given a local anaesthetic (2 to 3 drops; mix of 0.67% lidocaine [Alveda Pharmaceuticals, Toronto, ON, Canada] and 0.17% bupivacaine [Hospira, Inc., Montreal, QC, Canada]) on all incision areas (shaved and cleaned beforehand). At the end of each surgery, all mice were given a rehydrating 0.5mL s.c injection of warm saline solution (0.9% NaCl). All incisions for DEM castrations/ovariectomies (see below) were closed via MikRon wound clips (Autoclip, 9mm; MikRon Precision Inc., Gardena, CA). All mice were allowed to recover for (at least) 7 days (singly housed in a clean cage, with libitum food and water access, same housing parameters as described above) before being pair housed for the STFP, or tested in the ODT.

**Ovariectomy Surgery.** This surgery is described in detail in Clipperton-Allen et al., 2011 [4]. Briefly, for all adult anaesthetized DEM females, a 2cm dorsal incision was made on the skin of the lower back. Two lateral incisions (1cm each) were made on the muscles overlaying each ovary, which were then pulled out, the fallopian tubes were clamped, and the ovaries were resected (one at a time). The fallopian tubes were then reinserted into the muscles. The skin incision was closed using 1-2 wound clips (see above) [4].

**Castration Surgery.** Adult DEM males were first anesthetized, and then placed on their backs. A small ventral incision (1cm) was then made on the midline of the scrotum. Next, the skin was pulled back, and the lateral walls of the tunica were cut on the left and right sides (0.5cm each). One at a time, the testes were then individually pulled out, the spermatic cords were ligated (using hot sterile hemostatic clamps), and the testes were removed. The tubules and various blood vessels were then put back into the tunica, and the skin incision was closed with 1-2 wound clips (see above) [5].

**Cannulation Surgery.** See Phan et al., 2015 [6] for detailed procedures of this surgery. The skin on top of the skull of anesthetized OBS and ODT mice was removed, and via a stereotaxic frame (David Kopf instruments, CA) with atraumatic mouse ear bars, 26 gauge bilateral guide cannulae (Plastics One, HRS Scientific, Anjou, QC, Canada) were implanted into the skull aiming for the anterior DH, with respect to Bregma (flat skull position; AP= -1.7mm; Lat. = 1.5mm; DV [below the skull surface] = -1.3mm). Injectors (Plastics One, HRS Scientific, Anjou, QC, Canada) used during experimental procedures had a ventral coordinate of 2.3mm below the surface of the mouse skull. Anatomical coordinates were based on the mouse brain atlas of Paxinos and Franklin 2001. Jewellers screws (1.6mm; Plastics One, HRS Scientific, Anjou, QC, Canada) were inserted into the skull, and dental cement (Central Dental Ltd, Scarborough, ON, Canada) was used to keep the guide cannulae fixed in place. At the end of the surgery, dummy cannulae (Plastics One, HRS Scientific, Anjou, QC, Canada) were inserted into the guides to prevent any possible blockage or infection.

**Materials.** Custom-made perforated steel cage dividers (25.8 x .2 x 10.8 cm3; see Matta et al., 2017 for details) were used to keep same-sex mice physically separated during the three days of pair housing before STFP testing. DEM’s flavored diet consumption occurred in clean polyethylene cages (26 x 16 x 12 cm3) with glass jars (5cm H, 7.5cm W; Dyets, Bethlehem, PA) with stainless steel screw on lids equipped with collars. All jar lids had a hole that was 2.5cm in diameter for access to the food, and jars also contained a perforated stainless steel disk which was placed on top of the powdered food to thwart any possible spillage and make for more accurate intake measurements. All social interactions occurred in the OBS/DEM home cage with the cage divider removed and were recorded under infrared light using a JVC Everio camcorder (Mississauga, ON, Canada). Overhead recording was made possible using clear, glare-resistant, Plexiglas cage lids with air holes, which were used for all social interactions in the STFP and phases of the ODT. For choice testing in the STFP, OBS’s were placed into clean polyethylene cages (42.5 x 26.5 x 18.5cm3) which were equipped with two separate openings leading to stainless steel tunnels protruding at the front of the cage, where one tunnel led to a removable Plexiglas container filled with CIN, while the other tunnel led to a container filled with COC [see 1 and 7 for a detailed description of this equipment; Tecniplast, Varese, Italy]. A scale (Sartorius Analytical Balance, Sartorius Inc., United Kingdom) that was accurate to 0.01g was used to weigh all the DEM jars and OBS choice test feeding containers. All olfactory exposure phases for the ODT involved the use of glass mason jars with a height of 5.5cm and a diameter of 6.5cm. To give mice access to the odor of the flavored diets without being able to actually ingest it, the ODT mason jars also had screw-on lids (7cm diameter) equipped with a 0.5cm fine metal mesh grid (5.5cm diameter) [5]. Following all behavioral testing, all equipment/materials were washed with baking soda and odorless Alconox detergent powder, and left to air dry to eliminate any possible odor cues from trial to trial.

**Experimental Procedures**

On each day of testing, both female and male mice were always tested concurrently, and vaginal smears were taken from female mice to determine the phase of the estrus cycle. Researchers were blind to treatment, and drug order was counterbalanced.

**Olfactory Discrimination Task (ODT).** To determine whether the block of social learning in the STFP by DH raclopride could be accounted for by impaired olfactory discrimination between the CIN and COC diets female mice were repeatedly exposed (habituation) to the odor of one diet before a choice test between the two diets. Ten minutes before the first habituation, mice received an intrahippocampal microinfusion of raclopride at either 18 µg/µL or 20 µg/µL (the two doses that blocked social learning), or saline solution.

**Social Transmission of Food Preferences (STFP).** All OBSs were pair-housed with a same-sex DEM using perforated cage dividers that split the home cage into two sections (lengthwise) [see 5] for at least 3 days prior to behavioral testing. Perforated cage dividers were used when mice were pair-housed to prevent the DEM from chewing off the OBS’s cannula and headcap. Furthermore, perforations within the cage divider still allowed OBS-DEM dyads to become familiar and have sensory exposure to one another for the three-day period. The DEM/OBS’s side (right/left) was counterbalanced for each sex and dose throughout testing. Mice acclimatized to the experimental room over night, and to promote feeding, they were food deprived 12-14 hours prior to experimentation. Early in the dark period on the morning of testing under red light, DEMs were transferred to clean and empty cages for 1-hr where half the DEM’s had access to the CIN diet and the other half the COC diet. DEM’s food (CIN or COC) was counterbalanced for each sex/dose. Only OBS’s whose DEM consumed at least 0.1g of food were tested (no OBSs were excluded based on this criterion). After eating, the DEMs were returned to the home cage and allowed to socially interact (unobstructed, without a cage divider) with their respective same-sex OBS for 30-minutes. OBSs were then placed into the choice test cages where they had *ad libitum* access to both CIN and COC diets (and water) for 8 hours. The side each food was on (left or right) was counterbalanced for each sex/treatment throughout the experiment. Weighing’s were taken at 2, 4, 6, and 8 hours into choice testing using a scale precise to 0.01 g (Sartorius, UK) to determine the time-course of food preferences and total intake.

The doses selected were primarily based on our own behavioral pilot studies, as well as in vitro mouse studies that administered Raclopride within hippocampal or striatal brain slices [8-11], and behavioral mouse studies that infused raclopride into the striatum [12-17]. The ten-minute delay period was chosen based on previous behavioral mouse studies that used Raclopride systemically [1], in the NAc [12-15, 17], and in the dorsolateral striatum [16]. After the microinfusion ended, the injector was left in the brain for one additional minute to avoid any back-flow. Male and female OBSs were always tested on the same day, and the order in which the treatment was administered was counterbalanced for each sex, from trial to trial.

The social interactions were video recorded for later OBS behavioral analysis (The Observer Video software, Noldus Information Technology, Wageningen, Netherlands) that was scored by a trained researcher who was blind to the OBSs experimental group. Various social behaviors including social investigative actions (e.g., oronasal investigation, which is necessary for the STFP), agonistic behaviors (e.g., ritualized aggression), and non-social active behaviors (e.g., horizontal and vertical exploration) were examined during the social interactions (see Supplementary Tables S1 and S2 for full list) [2, 18, 19].

Grouped behaviors were also calculated from a combination of the single behaviors to examine whether drug treatment had an influence on aspects such as overall activity levels and social hierarchies.

**Figure S4.** Timeline for the intra-hippocampal Raclopride social transmission of food preferences (STFP) experiment. “DEM” stands for demonstrator mouse; “OBS” stands for observer mouse.

**Olfactory Discrimination Task (ODT).** Four habituation phases and one test phase were conducted consecutively in the home cage. Each phase lasted 5 minutes with ~1 minute in between each session. During habituation, mice were presented with 2 mason jars (with meshed lids) that each contained 15g of the same flavored food (either both CIN or both COC). During a choice test, one jar contained CIN and the other jar contained COC (i.e., one familiar, and one novel scented diet). A trained researcher quietly stood 1-meter away holding two stopwatches to measure the amount of time mice spent investigating (sniffing within 1-2mm) the meshed area of the jars (i.e., where the food odor was emitted). The following was counterbalanced for each treatment group: the flavored diet presented during the habituation phase, and the side the novel diet was placed (left/right) during the test phase [5]. Procedures including the length of food deprivation, and time delay between drug microinfusion and behavioral testing were conducted to mimic the STFP procedures.

**Figure S5.** Timeline for the intra-hippocampal Raclopride olfactory discrimination task (ODT). “Hab” stands for habituation phase.

**Estrous Phase Determination.** OBS vaginal smears were taken directly after the choice test was complete in the STFP, and after the test session in the ODT. To confirm that the ovariectomy surgeries were successful [see 20] DEM vaginal smears were also taken following the end of the 30-minute social interactions. To do this, females were first gently restrained, then cotton-tipped swabs were moistened with room temperature distilled water, inserted into the vagina, rotated 2-3 turns along the vaginal walls, and then applied onto clean glass microscope slides. To better visualize cells, microscope slides were later (at least 24 hours) stained with Giemsa (Sigma-Aldrich, Oakville, ON, Canada). Post-staining, slides were left to air-dry overnight in a fume hood, before being examined with a light microscope (3.2x magnification). Proestrus was determined as primarily nucleated epithelial cells, with very little to no leukocytes or cornified epithelial cells present. Estrus was determined by large amounts of cornified epithelial cells, though a few nucleated epithelial cells may have been present. Lastly, diestrus was determined as numerous leukocytes, with some occasional cornified or nucleated epithelial cells [2, 21, 22].

**Histology for determination of Cannulae Placement.** Following all behavioral testing, cannulated mice received a DH infusion of 1% Chicago blue dye in phosphate buffered saline (PBS) solution, under the same microinfusion parameters as during behavioral testing (see Figure S3). Brains were extracted 40 minutes after initiating dye infusions. This time point was chosen based on the time when OBS mice were given the food choice test following treatment infusions in the STFP paradigm. Brains were initially placed into 4% paraformaldehyde for approximately 1.5 to 2 weeks at 4°C. Following this, brains were then transferred into 30% sucrose in PBS solution for 3-5 days, or until the brains sank to the bottom a 50cc conical (also at 4°C). Brains were then kept frozen at -80 °C before being sectioned with a cryostat (coronal slices at 30μm; Leica CM 1850, Leica Microsystems, Richmond Hill, ON). Slices were mounted onto gelatin-coated glass microscope slides, and were later cover-slipped with DPX mounting solution. We found that the dye was mostly localized to the DH, which is consistent with our past DH studies [5, 23, 24]. Brains with incorrectly placed cannulae (as examined with a light microscope at 3.2x magnification) within the hippocampus were removed from all analyses (n = 13 mice removed across all treatment groups/sexes) [25].

**Data Handling***.* To determine whether social learned occurred in the STFP, a percent of CIN diet was calculated as ((CIN / CIN + COC) x100) for every OBS, at each time point (2, 4, 6 and 8 hours). A CIN preference score towards 0% implies that OBS’s preferred to eat COC, whereas a CIN preference score towards 100% implies that OBS’s preferred to eat CIN. A statistically significant difference between the CIN preference scores of OBSs who had a CIN DEM versus OBS’s who had a COC DEM (at a particular time point) denotes a socially learned food preference (for that treatment group).

To compare the strength of food preferences across different treatment groups, we also calculated a percent of DEM diet as (DEM / [DEM + NONDEM]) x100), for every OBS, at each time point (2, 4, 6 and 8 hours). A DEM preference score around 50% (chance levels) implies that OBS’s had no food preference, whereas a DEM preference score towards 100% implies that OBS’s preferred to eat their DEM’s food type [see 26].

A percent of CIN diet and percent of DEM diet ratio was calculated only if OBS’s ate at least 0.1g of food in total for each choice test time point. For this reason, there were often empty cells for certain time points, which decreased the sample size for the overall model. Accordingly, separate analyses were conducted on the CIN and DEM preference scores at each time point separately (2, 4, 6 and 8 hours), in addition to the analyses including the full 8 hours. The total amount of food consumed (CIN + COC) was also calculated for each OBS, at every time point.

For the ODT, a percent of novel diet investigation (NDI) value as (novel diet investigation time/total time investigating both diets) x100, and a total investigation value (novel diet investigation time + familiar diet investigation time) was calculated for all phases, for each mouse. Additionally, an average value of the four habituation NDI scores was calculated for every mouse. This average habituation value was expected to be around 50% (chance) because mice were investigating two jars containing the same diet. Since mice preferentially investigate novel over familiar stimuli [1], it was expected that the NDI scores during test would be significantly higher than the average habituation NDI scores.

The homogeneity of variance assumption is violated by ratio data. Accordingly, all ratio data was arcsin-transformed prior to analysis.

**Statistical Analysis.** Mixed model analyses of variance (ANOVAs) were conducted on the CIN preference score data. The between groups factors were: sex (male or female), DEM food (CIN or COC), treatment condition (saline, or Raclopride at 10, 14, 18, or 20 μg/μL), and the repeated measures factor was time (2, 4, 6 and 8 hours).

Mixed model ANOVAs were conducted on the DEM preference score data and total food intake data. The between groups factors were: sex (male or female), and treatment condition (saline, or Raclopride at 10, 14, 18, or 20 μg/μL), and the repeated measures factor was time (2, 4, 6 and 8 hours).

Food preferences within the STFP are mostly influenced by drug treatments within the early hours of testing (where the food preferences are most robust) [1, 2, 5, 27]. For this reason, mean comparisons were planned at these time points. Specifically, independent samples t-tests were conducted to compare the CIN preference scores of OBS’s who had a CIN fed DEM versus OBS’s who had a COC fed DEM at each time point, for each sex, and dose group. In doing so, we could address whether or not OBS’s showed a socially learned food preference. We also employed independent samples t-tests on the DEM preference scores between saline infused mice and Raclopride infused mice, at each time point, for each sex. In doing so, we could compare the strength of socially learned food preferences across different treatment groups. Planned one-sample t-tests comparing to 50% (chance) were also conducted on the DEM preference scores at each time point, for each sex, and dose group. These latter comparisons allowed the assessment of social learning in each group.

Mixed model ANOVAs were conducted on the duration, frequency and latency data derived from the single and grouped behaviors from the 30-minute social interactions in the STFP. The between groups factors in these models were: sex (male or female) and treatment condition (saline, or Raclopride at 10, 14, 18, or 20 μg/μL). We also performed planned independent samples t-tests on the saline versus Raclopride groups for each of the single and grouped behaviors for both sexes. We used non-parametric analyses (Kruskal–Wallis and Mann–Whitney U) when normality could not be achieved by ln transforming the data. Given that the duration, frequency and latency data yield largely consistent results, we only report the duration results below, and report the frequency/latency results only when different than the duration results.

To assess baseline sex differences during the social interactions, we also planned independent samples t-tests to compare the saline treated males to saline treated females for single and grouped behavioral data.

Mixed model ANOVAs were conducted on the NDI scores and ODT total investigation data. Additionally, planned paired samples t-tests were conducted comparing the averaged habituation NDI scores to the test NDI scores. A significant difference between these two values indicates that mice were able to discriminate between the two flavored diets. For the ODT, the between groups factor was treatment (saline, or Raclopride at 18 or 20 µg/µL), and the repeated measures factor was time (habituations to test).

The phase of the estrous cycle (proestrus, estrus, and diestrus) was one additional between groups factor that was applied to all models involving only female mice. This was done to establish whether or not the phases of the estrous cycle interacted with drug treatment to influence learning and/or behaviors.

Overall ANOVAs were conducted for all models to reduce the risk of committing type I errors, and all binary mean comparisons throughout were planned a priori to attain greater power and help reduce the total number of comparisons, thus reducing type I errors. Post-hoc multiple comparisons (e.g., Bonferroni) were not used here due to the fact that such corrections are very conservative, and often produce a larger chance of type II errors [see 7-10]. Indeed, many published neuroscience articles take the same statistical approach [see 31-36]. See Matta et al., 2017 [5] for further details about this statistical analysis decision. We applied the Greenhouse–Geisser correction for repeated measures. Statistical significance was set at p < .05. Non-significant values were not reported, except in the case of meaningful results or values, and a clear statistical trend (T = .05 < p < 0.1). All analyses were executed on SPSS version 20 (IBM Corp, Armonk, NY).

**RESULTS**

**Social Transmission of Food Preference Study: DH Raclopride Blocks Social Learning and Reduces the Strength of the Socially Acquired Food Preference Without Influencing Total Food Intake in Female but not Male Mice**

Separate ANOVAs conducted on the percent of CIN diets of OBSs at individual time points revealed a significant main effect of treatment at 2 hours (F(4, 197) = 3.42, *p* = .010), 4 hours (F(4, 201) = 2.38, *p* = .05), and 6 hours (F(4, 184) = 2.46, *p* = .047). There was also a significant main effect of DEM food at 2 hours (F(1, 197) = 90.11, *p* < .001), 4 hours (F(1, 201) = 19.13, *p* < .001), 6 hours (F(1, 184) = 12.21, *p* = .001), and 8 hours (F(1, 153) = 9.02, *p* = .003). Furthermore, there was a trend towards a treatment x DEM food interaction at 2 hours (F(4, 197) = 2.22, *p* = .068), and a trend towards a sex x DEM food interaction at 2 hours (F(1, 197) = 2.89, *p* = .091) and 8 hours (F(1, 153) = 2.96, *p* = .087). Interestingly, there was a significant main effect of sex at 8 hours (F(1, 153) = 3.90, *p* = .05). Thus, DH raclopride affected how the flavored food diet of the DEM drives the percent of CIN diets of OBS mice (i.e. raclopride affected the social acquisition of a food preference) in a time- and sex-dependent manner.

Separate RM ANOVAs conducted on the percent of CIN diets of females and males revealed a significant main effect of DEM food for males (F(1, 66) = 20.67, *p* < .001) and females (F(1, 66) = 7.29, *p* = .009). There was also a significant time x DEM food interaction for males (F(3, 198) = 2.92, *p* = .035) and a trend towards significance for females (F(3, 198) = 2.57, *p* = .055) for the full 8 hours. Furthermore, there was a significant main effect of DEM food at 2 hours (F(1, 80) = 14.31, *p* < .001) and a trend towards significance at 4 hours (F(1, 79) = 2.81, *p* = .098) for females, whereas males showed a significant main effect of DEM food at all time points (2 hours: F(1, 98) = 61.47, *p* < .001; 4 hours: F(1, 104) = 9.55, *p* = .003; 6 hours: F(1, 86) = 11.12, *p* = .001; 8 hours: F(1, 78) = 11.74, *p* = .001). Moreover, ANOVAs revealed a significant main effect of treatment at 2 hours (F(4, 80) = 3.28, *p* = .015) and 4 hours (F(4, 79) = 3.25, *p* = .016) for females, and for males at 6 hours (F(4, 86) = 2.99, *p* = .023) and 8 hours (F(4, 78) = 2.70, *p* = .037), while there was only a trend towards significance for males at 2 hours (F(4, 98) = 2.19, *p* = .076). Hence, DH raclopride influenced the percent of CIN diets of both male and female OBS’s for various time points during the choice test, and as a function of their DEMs food-type.

**Social Transmission of Food Preference Study: The Phase of the Estrous Cycle Affects how Raclopride Impacts Social Learning**

Similar to the CIN preference analysis, the RM ANOVA conducted on the percent of DEM diets of females revealed a significant time x estrous phase interaction (F(6, 189) = 2.81, *p* = .012) for the full 8 hours. There was also a trend towards a main effect of estrous phase at 6 hours (F(2, 93) = 2.64, *p* = .076). Hence, the estrous phase may have influenced the female percent of DEM diets in a time dependent manner. Unfortunately, mean comparisons did not yield any significances, likely due to the small number of females for each phase, per treatment group (See supplemental Table S3).

Interestingly, the RM ANOVA conducted only on female total intake showed a trend towards a time x estrous phase x treatment interaction (F(24, 267) = 1.52, *p* = .061) for the full 8 hours. Separate ANOVAs also showed a trend towards an estrous phase x treatment interaction at 2 hours (F(8, 89) = 1.98, *p* = .057) and 6 hours (F(8, 89) = 1.84, *p* = .079). Further mean comparisons did not reveal any significances, likely due to the small number of mice for each phase, per treatment group (Table S3).

**Table S3.** Sample sizes (*n*’s) for each estrous phase, per treatment group, for the social transmission of food preferences (STFP) DH Raclopride experiments.

| **Social transmission of food preferences (STFP)** | | | |
| --- | --- | --- | --- |
|  | Proestrus | Estrus | Diestrus |
| Saline | *n* = 4 | *n* = 3 | *n* = 16 |
| Raclopride at 10 μg/μL | *n* = 2 | *n* = 4 | *n* = 18 |
| Raclopride at 14 μg/μL | *n* = 2 | *n* = 9 | *n* = 12 |
| Raclopride at 18 μg/μL | *n* = 5 | *n* = 5 | *n* = 14 |
| Raclopride at 20 μg/μL | *n* = 4 | *n* = 5 | *n* = 15 |

**Total Food Intake**

The RM ANOVA conducted on the total intakes of OBS mice revealed a significant main effect of time (F(3, 648) = 76.45, p < .001). Separate RM ANOVAs revealed a significant main effect of time for both sexes (males: F(3, 324) = 38.97, p < .001; females: F(3, 267) = 19.07, p < .001). There were no significant main effects of treatment or sex, and no treatment x sex interactions (or interactions with time) for the full 8 hours or at separate time points for either sex. Hence, the social learning impairment by DH raclopride found in this study is not secondary to changes in feeding.

**Figure S6.** Total food intake (1% ground cinnamon [CIN] + 2% ground cocoa [COC]) for observer (OBS) female (A) and male (B) mice that received a single DH microinfusion of either saline vehicle (white circles; *n* = 23 females and *n* = 24 males), or the D2-type dopamine receptor antagonist Raclopride at 10 μg/μL (black triangles; *n* = 24 females and *n* = 23 males), 14 μg/μL (black circles; *n* = 23 females and *n* = 24 males), 18 μg/μL (black squares; *n* = 24 females and *n* = 24 males) or 20 μg/μL (black diamonds; *n* = 24 females and *n* = 23 males) 10-minutes prior to the 30-minute social interaction with a same-sex demonstrator (DEM) mouse that recently consumed either a CIN or COC flavored diet. Total food intakes are displayed at 2, 4, 6 and 8 hours into the OBS choice test. There were no significant effects of DH raclopride on total food intakes for either sex. Data are presented as mean **±** SEM.

**Effects of DH Raclopride on Behavior During the Social Interactions in the Social Transmission of Food Preference Study**

While total activity was only reduced by DH raclopride in female mice, total social behavior was reduced in both sexes. This was paralleled by an increase in the amount of time spent engaging in non-social non-locomotor behaviors (such as solitary inactivity) in both females and males.

DH raclopride treatment reduced agonistic and investigatory behaviors in both sexes, however, there was a larger effect on agonistic behaviors in males, and on social investigatory behaviors in females (i.e., the behavior that each sex performs more of, *in general*). Specifically, male overt agonistic behaviors (e.g., boxing and agonistic behavior delivered) were reduced. On the other hand, female social investigatory behaviors (e.g., body and anogenital investigation) were reduced.

DH raclopride led to a reduced dominance score for both males and females, which indicates that they were both more subordinate during the social interactions than saline treated control mice.

Notably, the social learning impairment found in females due to DH raclopride was not accompanied by any changes in the time spent engaging in oronasal investigation. This suggests that the social learning impairment found in this study could not be explained by any changes in the amount of exposure to the food odor emitted by the DEM.

ANOVAs revealed a significant main effect of sex for a reduction by DH raclopride of total activity (F(1, 226) = 5.601, *p* = .019). This could be partially explained by the significant main effect of sex for non-social locomotor behaviors (F(1, 226) = 4.191, *p* = .042) which in turn could be partially accounted for by the significant main effect of sex for digging (F(1, 226) = 4.709, *p* = .031). There was also a trend towards a sex x drug interaction for vertical exploration (F(4, 226) = 2.036, *p* = .09). Additionally, a trend towards a main effect of sex for horizontal exploration (F(1, 226) = 3.629, *p* = .058) was found.

There were various sex-mediated inhibitory effects of DH raclopride on agonistic behaviors. For instance, there was a significant main effect of sex (F(1, 226) = 28.878, *p* < .001) and a trend towards a sex x drug interaction (F(4, 226) = 2.063, *p* = .087) for the frequency of total agonistic behaviors. This effect could be partially explained by the significant main effect of sex (F(1, 226) = 42.693, *p* < .00)] and sex x drug interaction (F(4, 226) = 2.377, *p* = .05) for boxing. Moreover, there was a significant main effect of sex for open aggression (F(1, 226) = 19.046, *p* < .001). ANOVAs also revealed a trend towards a main effect of sex for dominant behavior (F(1, 226) = 3.431, *p* = .065), and a significant main effect of sex (F(1, 226) = 39.695, *p* < .001) and a trend towards a sex x drug interaction (F(4, 226) = 2.315, *p* = .058) for the frequency of agonistic behavior delivered. In line with this, ANOVAs revealed a significant main effect of sex (F(1, 226) = 43.414, *p* < .001) and a trend towards a sex x drug interaction (F(4, 226) = 2.151, *p* = .075) for the frequency of attacks delivered. There was also a significant main effect of sex for agonistic behavior received (F(1, 226) = 16.672, *p* < .001), and avoid frequency (F(1, 226) = 5.472, *p* = .02). Additionally, ANOVAs showed a significant main effect of sex (F(1, 226) = 16.118, *p* < .001) and sex x drug interaction (F(4, 226) = 2.451, *p* = .047) for submissive behavior. As a result, these effects led to a significant main effect of sex for dominance score (F(1, 226) = 17.411, *p* < .001). Specifically, total agonistic behaviors were greater for males than females, whereby males spent more time engaging in boxing, open aggression, and dominant behavior than females. Males also more frequently delivered agonistic behaviors (they attacked more often) than females. Conversely, females received more agonistic behaviors, and were more submissive than males. The dominance score was therefore greater for males than females. Hence, males engaged in agonistic-type behaviors to a greater extent than females (also see the below section on sex differences in mice infused with saline).

There were also many sex-mediated social-investigatory effects of DH raclopride. ANOVAs revealed a significant main effect of sex (F(1, 226) = 5.744, *p* = .017) and sex x drug interaction (F(4, 226) = 2.825, *p* = .026) for social investigation. This could be partly accounted for by the significant main effect of sex (F(1, 226) = 9.707, *p* = .002) and sex x drug interaction (F(4, 226) = 3.243, *p* = .013) for body investigation as well as the significant main effect of sex (F(1, 226) = 4.144, *p* = .043) and sex x drug interaction (F(4, 226) = 2.818, *p* = .026) for anogenital investigation. Furthermore, there was a trend towards a main effect of sex for approaching and/or attending to the DEM (F(1, 226) = 3.429, *p* = .065). Additionally, ANOVAs showed a significant sex x drug interaction for the frequency of following the DEM (F(4, 226) = 3.557, *p* = .008). There was also a significant main effect of sex for the frequency of social inactivity (F(1, 226) = 4.018, *p* = .046). Specifically, females spent longer performing social investigation, whereby they spent more time engaging in body and anogenital investigation, and more frequently followed the DEM than males. Thus, social-investigatory behaviors were greater for female mice (also see the below section on sex differences in mice infused with saline).

ANOVAs revealed a significant main effect of sex for non-social non-locomotor behaviors (F(1, 226) = 8.423, *p* = .004). This could be partly explained by the significant main effect of sex for solitary inactivity (F(1, 226) = 8.110, *p* = .005).

Separate ANOVAs run on only females or males revealed that female total activity was significantly reduced (F (4, 113) = 4.26, *p* = 0.003) by all doses of raclopride (10 μg/μL: *t*(45) = 3.332, *p* = 0.002; 14 μg/μL: *t*(44) = 2.606, *p* = 0.012; 18 μg/μL: *t*(45) = 3.827, *p* < .001; 20 μg/μL: *t*(45) = 3.163, *p* = 0.003). This could be partially explained by the finding that female total social behavior was significantly reduced (F (4, 113) = 4.158, *p* = 0.004) by all doses of raclopride (10 μg/μL: *t*(45) = 2.205, *p* = 0.033; 14 μg/μL: *t*(44) = 2.922, *p* = 0.005; 18 μg/μL: *t*(45) = 3.497, *p* = 0.001; 20 μg/μL: *t*(45) = 3.15, *p* = 0.003).

While male total activity was unaffected by drug treatment, male total social behavior was significantly reduced (F (4, 113) = 7.485, *p* < .001) by all doses of raclopride (10 μg/μL: *t*(45) = 4.687, *p* < .001; 14 μg/μL: *t*(46) = 4.454, *p* < .001; 18 μg/μL: *t*(46) = 2.835, *p* = 0.007; 20 μg/μL: *t*(45) = 4.14, *p* < .001).

**Figure S7.** Active behaviors (in sec) for observer (OBS) female [A, C] and male [B, D] mice that received a single DH microinfusion of either saline vehicle (*n* = 23 females and *n* = 24 males), or the D2-type dopamine receptor antagonist raclopride at 10 μg/μL (*n* = 24 females and *n* = 23 males), 14 μg/μL (*n* = 23 females and *n* = 24 males), 18 μg/μL (*n* = 24 females and *n* = 24 males) or 20 μg/μL (*n* = 24 females and *n* = 23 males). Infusions were administered 10 minutes prior to a 30 minute social interaction where OBS mice had the opportunity to acquire a food preference from a same-sex demonstrator (DEM) mouse that recently consumed either a 1% ground cinnamon (CIN) or 2% ground cocoa (COC) flavored diet. DH raclopride effects on female [A] and male [B] total activity, and female [C] and male [D] total social behavior are shown. There were no significant effects of DH raclopride on male total activity [B]. Data are presented as mean **+** SEM. **p* < 0.05, ***p* < 0.01, ****p* < 0.001, in comparison to saline infused control animals.

It is interesting to note that for both females and males, all non-social locomotor behaviors (horizontal exploration, vertical exploration, and dig) were unaffected by DH raclopride.

The frequency of male total agonistic behaviors was significantly reduced (F (4, 113) = 2.602, *p* = 0.04) by the two highest doses of raclopride (18 μg/μL: *t*(46) = 2.087, *p* = .042; 20 μg/μL: *t*(45) = 2.604, *p* = .012). An overall reduction in agonistic behaviors delivered, and an increase in submissive behaviors and agonistic behaviors received resulted in a significantly reduced male dominance score (F (4, 113) = 7.163, *p* < .001) by all doses of raclopride (10 μg/μL: *t*(45) = 4.907, *p* < .001; 14 μg/μL: *t*(46) = 5.215, *p* < .001; 18 μg/μL: *t*(46) = 3.912, *p* < .001; 20 μg/μL: *t*(45) = 4.163, *p* < .001). Specifically, male dominant behavior was significantly reduced (χ^2^(4) = 19.106, *p* = .001) by all doses of raclopride (10 μg/μL: *U* = 118.5, z = -3.355, *p* = .001; 14 μg/μL: *U* = 113.5, z = -3.602, *p* < .001; 18 μg/μL: *U* = 127.5, z = -3.317, *p* = .001; 20 μg/μL: *U* = 117, z = -3.386, *p* = .001). Additionally, male boxing was significantly reduced (F (4, 113) = 2.413, *p* = 0.05) by 20 μg/μL (*t*(45) = 2.434, *p* = 0.019) while raclopride at 18 μg/μL only trended towards significance (*t*(46) = 1.807, *p* = 0.077). Furthermore, male submissive behavior was significantly increased (F (4, 113) = 2.585, *p* = 0.041) by all doses of raclopride (10 μg/μL: *t*(45) = -2.609, *p* = 0.012; 14 μg/μL: *t*(46) = -2.841, *p* = 0.007; 18 μg/μL: *t*(46) = -2.737, *p* = 0.009; 20 μg/μL: *t*(45) = -2.824, *p* = 0.007). In line with this, male agonistic behavior received was significantly increased (χ^2^(4) = 20.101, *p* < .001) by all doses of raclopride (10 μg/μL: *U* = 383, z = 2.285, *p* = .022; 14 μg/μL: *U* = 454.5, z = 3.441, *p* = .001; 18 μg/μL: *U* = 422.5, z = 2.786, *p* = .005; 20 μg/μL: *U* = 482.5, z = 4.399, *p* < .001). Moreover, male agonistic behavior delivered was significantly reduced (χ^2^(4) = 24.111, *p* < .001) by all doses of raclopride (10 μg/μL: *U* = 98, z = -3.788, *p* < .001; 14 μg/μL: *U* = 85, z = -4.186, *p* < .001; 18 μg/μL: *U* = 122, z = -3.423, *p* = .001; 20 μg/μL: *U* = 90, z = -3.958, *p* < .001). The frequency of male attacks delivered (F (4, 113) = 2.157, *p* = 0.078) and open aggression (F (4, 113) = 2.136, *p* = 0.081) also trended towards a decrease. Collectively, these results suggest that DH raclopride reduced many male-typical agonistic-related behaviors during the social interactions.

**Figure S8.** Agonistic behaviors for observer (OBS) male mice that received a single DH microinfusion of either saline vehicle (*n* = 24), or the D2-type dopamine receptor antagonist raclopride at 10 μg/μL (*n* = 23), 14 μg/μL (*n* = 24), 18 μg/μL (*n* = 24) or 20 μg/μL (*n* = 23). Infusions were administered 10 minutes prior to a 30 minute social interaction where OBS mice had the opportunity to acquire a food preference from a same-sex demonstrator (DEM) mouse that recently consumed either a 1% ground cinnamon (CIN) or 2% ground cocoa (COC) flavored diet. DH raclopride effects on male total agonistic behaviors [A], dominance score [B], dominant behavior [C], boxing [D], submissive behavior [E], agonistic behavior received [F], and agonistic behavior delivered [G] are shown. Data are presented as mean **+** SEM. **p* < 0.05, ***p* < 0.01, ****p* < 0.001, T = 0.05 < *p* < 0.1, in comparison to saline infused control animals.

In addition to agonistic behaviors, other social behaviors were influenced by DH raclopride in males. That is, male social inactivity, the social behavior that involves the least amount of locomotion, was significantly reduced (F (4, 113) = 2.727, *p* = 0.033) by 10 μg/μL of raclopride (*t*(45) = 2.672, *p* = 0.01). Moreover, male social investigation was significantly reduced (F (4, 113) = 3.319, *p* = 0.013) by the two lowest doses (10 μg/μL: *t*(45) = 2.273, *p* = 0.028; 14 μg/μL: *t*(46) = 3.393, *p* = 0.001), while the highest dose of Raclopride trended towards significance (*t*(45) = 1.737, *p* = 0.089). This could be partially explained by the finding that male body investigation was significantly reduced (F (4, 113) = 3.005, *p* = 0.021) by the two lowest doses of Raclopride (10 μg/μL: *t*(45) = 2.479, *p* = 0.017; 14 μg/μL: *t*(46) = 3.393, *p* = 0.001). Furthermore, male anogenital investigation was significantly reduced (F (4, 113) = 4.51, *p* = 0.002) by the two lowest doses, and the highest dose of Raclopride (10 μg/μL: *t*(45) = 2.214, *p* = 0.032; 14 μg/μL: *t*(46) = 4.202, *p* < .001; 20 μg/μL: *t*(45) = 2.967, *p* = 0.005). In line with these findings, the frequency of males following the DEM also trended towards a reduction (F (4, 113) = 2.174, *p* = 0.076). Thus, DH raclopride decreased social inactive and various social-investigatory behaviors in males.

The general reduction in total social behaviour in females may be partly accounted for by the significant decrease in female social investigation (F (4, 113) = 7.618, *p* < .001) by all doses of raclopride (10 μg/μL: *t*(45) = 3.261, *p* = 0.002; 14 μg/μL: *t*(44) = 3.865, *p* < .001; 18 μg/μL: *t*(45) = 4.814, *p* < .001; 20 μg/μL: *t*(45) = 4.197, *p* < .001). This could be partially explained by the finding that female body investigation was significantly reduced (F (4, 113) = 8.64, *p* < .001) by all doses of raclopride (10 μg/μL: *t*(45) = 3.338, *p* = 0.002; 14 μg/μL: *t*(44) = 3.924, *p* < .001; 18 μg/μL: *t*(45) = 5.251, *p* < .001; 20 μg/μL: *t*(45) = 4.186, *p* < .001). Moreover, female anogenital investigation was significantly reduced (F (4, 113) = 7.911, *p* < .001) by all doses of raclopride (10 μg/μL: *t*(45) = 3.317, *p* = 0.002; 14 μg/μL: *t*(44) = 3.155, *p* = 0.003; 18 μg/μL: *t*(45) = 5.115, *p* < .001; 20 μg/μL: *t*(45) = 3.998, *p* < .001). Following the DEM was also significantly reduced in females (F (4, 113) = 4.774, *p* = 0.001) by all doses of raclopride (10 μg/μL: *t*(45) = 2.08, *p* = 0.043; 14 μg/μL: *t*(44) = 2.615, *p* = 0.012; 18 μg/μL: *t*(45) = 4.152, *p* < .001; 20 μg/μL: *t*(45)= 2.844, *p* = .007). Therefore, drug treatment decreased many social-investigatory behaviors in female mice.

**Figure S9.** Social investigatory behaviors (in sec) for observer (OBS) female [A, C, E, G, I] and male [B, D, F, H, J] mice that received a single DH microinfusion of either saline vehicle (*n* = 23 females and *n* = 24 males), or the D2-type dopamine receptor antagonist raclopride at 10 μg/μL (*n* = 24 females and *n* = 23 males), 14 μg/μL (*n* = 23 females and *n* = 24 males), 18 μg/μL (*n* = 24 females and *n* = 24 males) or 20 μg/μL (*n* = 24 females and *n* = 23 males). Infusions were administered 10 minutes prior to a 30-minute social interaction where OBS mice had the opportunity to acquire a food preference from a same-sex demonstrator (DEM) mouse that recently consumed either a 1% ground cinnamon (CIN) or 2% ground cocoa (COC) flavored diet. DH raclopride effects on female and male social investigation [A, B], oronasal investigation [C, D], body investigation [E, F], anogenital investigation [G, H], and following the DEM [I, J] are shown. There were no significant effects of DH raclopride on either female [C] or male [D] oronasal investigation, or following the DEM for males [J]. Data are presented as mean **+** SEM. **p* < 0.05, ***p* < 0.01, ****p* < 0.001, T = 0.05 < *p* < 0.1, in comparison to saline infused control animals.

In addition to social-investigatory behaviors, DH raclopride influenced agonistic behaviors in females. Female total agonistic behaviors trended towards a decrease (F (4, 113) = 1.995, *p* = 0.1). Notably, reduced dominant behavior and agonistic behavior delivered resulted in the female dominance score to be significantly reduced (F (4, 113) = 5.241, *p* = 0.001) by all doses of raclopride (10 μg/μL: *t*(45) = 3.726, *p* = 0.001; 14 μg/μL: *t*(44) = 2.028, *p* = 0.049; 18 μg/μL: *t*(45) = 3.403, *p* = 0.001; 20 μg/μL: *t*(45) = 2.752, *p* = 0.009). Specifically, this could be partly explained by the finding that female agonistic behavior delivered was significantly reduced (F (4, 113) = 6.677, *p* < .001) by all doses of raclopride (10 μg/μL: *t*(45) = 2.88, *p* = 0.006; 14 μg/μL: *t*(44) = 2.437, *p* = 0.019; 18 μg/μL: *t*(45) = 4.616, *p* < .001; 20 μg/μL: *t*(45) = 3.981, *p* < .001). Furthermore, female dominant behavior was significantly reduced (F (4, 113) = 4.278, *p* = 0.003) at the lowest, and two highest doses of raclopride, while 14 μg/μL only approached significance (10 μg/μL: *t*(45) = 2.395, *p* = 0.021; 14 μg/μL: *t*(44) = 1.842, *p* = 0.072; 18 μg/μL: *t*(45) = 3.789, *p* < .001; 20 μg/μL: *t*(45) = 3.408, *p* = .001). In addition, female submissive behavior (F (4, 113) = 2.023, *p* = 0.096) and agonistic behavior received (F (4, 113) = 2.001, *p* = 0.099) trended towards an increase. Thus, various agonistic-related behaviors were reduced by drug treatment in females.

**Figure S10.** Agonistic behaviors (in sec) for observer (OBS) female mice that received a single DH microinfusion of either saline vehicle (*n* = 23), or the D2-type dopamine receptor antagonist raclopride at 10 μg/μL (*n* = 24), 14 μg/μL (*n* = 23), 18 μg/μL (*n* = 24) or 20 μg/μL (*n* = 24). Infusions were administered 10 minutes prior to a 30-min social interaction where OBS mice had the opportunity to acquire a food preference from a same-sex demonstrator (DEM) mouse that recently consumed either a 1% ground cinnamon or 2% ground cocoa flavored diet. DH raclopride effects on female agonistic behavior delivered [A], dominant behavior [B], and dominance score [C] are shown. Data are presented as mean **+** SEM. **p* < 0.05, ***p* < 0.01, ****p* < 0.001, T = 0.05 < *p* < 0.1, in comparison to saline infused control animals.

Female non-social behaviors were significantly increased (F (4, 113) = 3.262, *p* = 0.014) by all doses of raclopride (10 μg/μL: *t*(45) = -2.569, *p* = 0.014; 14 μg/μL: *t*(44) = -2.93, *p* = 0.005; 18 μg/μL: *t*(45) = -3.293, *p* = 0.002; 20 μg/μL: *t*(45) = -2.558, *p* = 0.014).

Likewise, male non-social behaviors were significantly increased (F (4, 113) = 4.913, *p* = 0.001) by all doses of raclopride (10 μg/μL: *t*(45) = -4.318, *p* < .001; 14 μg/μL: *t*(46) = -2.939, *p* = 0.005; 18 μg/μL: *t*(46) = -2.389, *p* = 0.021; 20 μg/μL: *t*(45) = -3.561, *p* = 0.001).

**Figure S11.** Non-social behaviors (in sec) for observer (OBS) female [A] and male [B] mice that received a single DH microinfusion of either saline vehicle (*n* = 23 females and *n* = 24 males), or the D2-type dopamine receptor antagonist raclopride at 10 μg/μL (*n* = 24 females and *n* = 23 males), 14 μg/μL (*n* = 23 females and *n* = 24 males), 18 μg/μL (*n* = 24 females and *n* = 24 males) or 20 μg/μL (*n* = 24 females and *n* = 23 males). Infusions were administered 10 minutes prior to a 30-minute social interaction where OBS mice had the opportunity to acquire a food preference from a same-sex demonstrator (DEM) mouse that recently consumed either a 1% ground cinnamon (CIN) or 2% ground cocoa (COC) flavored diet. Data are presented as mean **+** SEM. **p* < 0.05, ***p* < 0.01, ****p* < 0.001, in comparison to saline infused control animals.

Female non-social non-locomotor behaviors were significantly increased (F (4, 113) = 4.911, *p* = 0.001) by all doses of raclopride (10 μg/μL: *t*(45) = -3.731, *p* = 0.001; 14 μg/μL: *t*(44) = -2.517, *p* = 0.016; 18 μg/μL: *t*(45) = -3.726, *p* = 0.001; 20 μg/μL: *t*(45) = -3.127, *p* = 0.003). This effect could be partly explained by the finding that female solitary inactivity was significantly increased (F (4, 113) = 10.191, *p* < .001) by all doses of Raclopride (10 μg/μL: *t*(45) = -5.243, *p* < .001; 14 μg/μL: *t*(44) = -4.469, *p* < .001; 18 μg/μL: *t*(45) = -6.065, *p* < .001; 20 μg/μL: *t*(45) = -6.069, *p* < .001). In addition, female self-grooming trended towards a reduction (F (4, 113) = 1.931, *p* = 0.10). Thus, the overall decrease in female total activity by DH raclopride was paralleled by an increase in non-social non-locomotor behaviors.

Similarly, male non-social non-locomotor behaviors were significantly increased (F (4, 113) = 2.414, *p* = 0.05) by the three highest doses of raclopride (14 μg/μL: *t*(46) = -2.023, *p* = 0.049; 18 μg/μL: *t*(46) = -2.269, *p* = 0.028; 20 μg/μL: *t*(45) = -2.992, *p* = 0.004). These results could be partly explained by the finding that male solitary inactivity was significantly increased (χ^2^(4) = 28.755, *p* < .001) by all doses of Raclopride (10 μg/μL: *U* = 462, z = 3.958, *p* < .001; 14 μg/μL: *U* = 472, z = 3.794, *p* < .001; 18 μg/μL: *U* = 457, z = 3.485, *p* < .001; 20 μg/μL: *U* = 505, z = 4.873, *p* < .001). Moreover, male self-grooming was significantly reduced (F (4, 113) = 3.06, *p* = 0.02) by all doses of raclopride (10 μg/μL: *t*(45) = 2.48, *p* = 0.017; 14 μg/μL: *t*(46) = 2.215, *p* = 0.032; 18 μg/μL: *t*(46) = 2.366, *p* = 0.022; 20 μg/μL: *t*(45) = 2.474, *p* = 0.017). Thus, DH raclopride treatment resulted in an increase in the time spent engaging in non-social non-locomotor behaviors in males.

**Figure S12.** Non-social non-locomotor behaviors (in sec) for observer (OBS) female [A, C, E] and male [B, D, F] mice that received a single DH microinfusion of either saline vehicle (*n* = 23 females and *n* = 24 males), or the D2-type dopamine receptor antagonist raclopride at 10 μg/μL (*n* = 24 females and *n* = 23 males), 14 μg/μL (*n* = 23 females and *n* = 24 males), 18 μg/μL (*n* = 24 females and *n* = 24 males) or 20 μg/μL (*n* = 24 females and *n* = 23 males). Infusions were administered 10 minutes prior to a 30 minute social interaction where OBS mice had the opportunity to acquire a food preference from a same-sex demonstrator (DEM) mouse that recently consumed either a 1% ground cinnamon (CIN) or 2% ground cocoa (COC) flavored diet. DH raclopride effects on female and male non-social non-locomotor behaviors [A, B], solitary inactivity [C, D], and self-grooming [E, F] are shown. There were no significant effects of DH raclopride on female self-grooming [E]. Data are presented as mean **+** SEM. **p* < 0.05, ***p* < 0.01, ****p* < 0.001, in comparison to saline infused control animals.

No other significances were found. Notably, DH raclopride did not produce any abnormal stereotypies. No estrous cycle effects were found on the behaviors during the social interactions. This was likely due to the small number of females in each phase, per treatment group (Table S3).

**Table S4.** Summarized DH raclopride effects on grouped behaviors during the 30min social interactions for the STFP experiment.

|  | **Females** | **Males** | **Saline treated sex difference** |
| --- | --- | --- | --- |
| **Total Activity** | ↓ | ⎯ | ⎯ |
| **Total Social Behavior** | ↓ | ↓ | ⎯ |
| **Agonistic Behavior Delivered** | ↓ | ↓ | ^[F]^ Females < Males |
| **Agonistic Behavior Received** | ⇡ | ↑ | Females > Males |
| **Total Agonistic Behaviors** | ⇣ | ^[F]^↓ | Females < Males |
| **Dominance Score** | ↓ | ↓ | Females < Males |
| **Social Investigation** | ↓ | ↓ | Females > Males |
| **Non-social Behaviors** | ↑ | ↑ | ⎯ |
| **Non-social Locomotor Behaviors** | ⎯ | ⎯ | ⎯ |
| **Non-social Non-locomotor Behaviors** | ↑ | ↑ | ⎯ |

⎯ no significant drug effect; ↓ significant decrease; ↑ significant increase; ⇣ trend towards decrease; ⇡ trend towards increase. Unless otherwise specified with an “F” (which indicates frequency effect), all effects are on duration.

**Table S5.** Summarized DH raclopride effects on single behaviors during the 30min social interactions for the STFP experiment.

|  | **Females** | **Males** | **Saline treated sex difference** |
| --- | --- | --- | --- |
| **Social Behaviors** | | | |
| **Following the DEM** | ↓ | ^[F]^⇣ | ^[F]^ Females > Males |
| **Dominant Behavior** | ↓ | ↓ | ^[T]^ Females < Males |
| **Attacks Delivered** | ⎯ | ^[F]^⇣ | ^[F]^ Females < Males |
| **Boxing** | ⎯ | ↓ | Females < Males |
| **Open Aggression** | ⎯ | ^[F]^⇣ | Females < Males |
| **Avoidance of DEM** | ⎯ | ⎯ | ⎯ |
| **Submissive Behavior** | ⇡ | ↑ | Females > Males |
| **Attacks Received** | ⎯ | ⎯ | ⎯ |
| **Defensive Upright Posturing** | ⎯ | ⎯ | ⎯ |
| **Social Inactivity** | ⎯ | ↓ | ⎯ |
| **Oronasal Investigation** | ⎯ | ⎯ | ⎯ |
| **Body Investigation** | ↓ | ↓ | Females > Males |
| **Anogenital investigation** | ↓ | ↓ | ^[T]^ Females > Males |
| **Stretched Approach** | ⎯ | ⎯ | ⎯ |
| **Approaching and/or Attending to the DEM** | ⎯ | ⎯ | ⎯ |
| **Non-social Behaviors** | | | |
| **Horizontal Exploration** | ⎯ | ⎯ | ⎯ |
| **Vertical Exploration** | ⎯ | ⎯ | ⎯ |
| **Digging** | ⎯ | ⎯ | ⎯ |
| **Abnormal Stereotypies** | ⎯ | ⎯ | ⎯ |
| **Solitary Inactivity** | ↑ | ↑ | ^[F] [T]^ Females > Males |
| **Self-Grooming** | ⇣ | ↓ | ⎯ |

⎯ no significant drug effect; ↓ significant decrease; ↑ significant increase; ⇣ trend towards decrease; ⇡ trend towards increase; “T” indicates statistical trend (.05 < *p* < 0.1). Unless otherwise specified with an “F” (which indicates frequency effect), all effects are on duration.

**Sex Differences in Saline Infused Mice During the Social Interactions in the STFP**

Males were more agonistic than females, as they boxed more, delivered more attacks, and had a higher dominance score. On the other hand, social investigation type behaviors were greater for female mice, as they engaged in more body and anogenital investigation than males.

Males had significantly greater (*t*(45) = -2.38, *p* = 0.022) total agonistic behaviors than females. This could be partly explained by the finding that males spent significantly more time boxing (*t*(45) = -3.163, *p* = 0.003), engaged in open aggression (*t*(45) = -2.5, *p* = 0.016), and trended towards spending more time engaging in dominant behavior (*t*(45) = -1.846, *p* = 0.072) than females. Moreover, the frequency of agonistic behavior delivered was significantly greater (*t*(45) = -3.302, *p* = 0.002) for males than females. Specifically, the frequency of attacks delivered was significantly greater (*U* = 414, z = 3.833, *p* < .001) for males. On the other hand, females spent significantly more time engaging in submissive behavior (*U* = 77.5, z = -4.348, *p* < .001), and received significantly more agonistic behaviors (*U* = 83, z = -4.114, *p* < .001) than males. Correspondingly, males had a significantly higher (*t*(45) = -3.369, *p* = 0.002) dominance score than females. Hence, males displayed more agonistic-related behaviors than females.

Females spent significantly (*t*(45) = 2.772, *p* = 0.008) longer performing social investigation than males. This could be partially accounted for by the finding that females spent significantly more time engaging in body investigation (*t*(45) = 3.203, *p* = 0.002), and trended towards spending more time engaging in anogenital investigation (*t*(45) = 1.957, *p* = 0.057) than males. Moreover, the frequency of following the DEM was significantly greater (*t*(45) = 2.518, *p* = 0.015) for females than males. There was also a trend towards females more frequently engaging in solitary inactivity (*t*(45) = 1.772, *p* = 0.083). Hence, saline treated females displayed more social-investigatory type behaviors than saline treated males.

**Figure S13.** Baseline sex differences for observer (OBS) mice that received a single DH microinfusion of saline vehicle (*n* = 23 females [black bars] and *n* = 24 males [white bars]). Infusions were administered 10 minutes prior to a 30 minute social interaction where OBS mice had the opportunity to acquire a food preference from a same-sex demonstrator (DEM) mouse that recently consumed either a 1% ground cinnamon (CIN) or 2% ground cocoa (COC) flavored diet. Sex differences are shown for various grouped and single behaviors for duration [A] and frequency [B] data. “Ago Rec” stands for agonistic behavior received; “Tot Ago” stands for total agonistic behaviors; “Dom Score” stands for dominance score; “Soc Inves” stands for social investigation; “Dominant Behav” stands for dominant behavior; “Open Aggr” stands for open aggression; “Submissive” stands for submissive behavior; “Body Inves” stands for body investigation; “Anogenital Inves” stands for anogenital investigation; “Ago Del” stands for agonistic behavior delivered; “Follow” stands for following the DEM; “Attacks Del” stands for attacks delivered; “Sol Inactivity” stands for solitary inactivity. Data are presented as mean **+** SEM. **p* < 0.05, ***p* < 0.01, ****p* < 0.001, T = 0.05 < *p* < 0.1, saline infused females versus saline infused males.

**Effects of DH Raclopride on Female Olfactory Discrimination**

The results of the NDI scores suggest that female mice infused with raclopride at 18 µg/µL and 20 µg/µL, the two doses that blocked the STFP in females, could discriminate between the two flavored diets used in the STFP choice test. Hence, the social learning impairment by DH raclopride found in this study cannot be directly explained by changes in olfactory discrimination.

The RM ANOVA run on the NDI scores for the average of the four habituation sessions and test session revealed a significant main effect of time (F(1, 32) = 51.457, *p* < .001), but there was no significant time x treatment interaction, and no main effect of treatment. Notably, planned paired samples *t*-tests conducted on the NDI scores of the average of the four habituation sessions versus test session further revealed that the NDI scores during test were significantly higher than during habituation for all groups tested (saline: *t*(11) = -5.232, *p* < .001; raclopride at 18 µg/µL: *t*(10) = -2.592, *p* = .027; raclopride at 20 µg/µL: *t*(11) = -5.458, *p* < .001). Thus, females infused with the two highest doses of raclopride (that blocked the STFP) could discriminate between the two foods used during the choice test in the social learning study.

The RM ANOVA conducted on the total investigation data revealed no significant time x treatment interaction, and no main effect of treatment, but there was a significant main effect of time (F(4, 128) = 8.793, *p* < .001). Independent samples *t*-tests further revealed that during the first habituation session, saline infused animals had significantly higher investigation times than mice infused with raclopride at 18 µg/µL (*t*(21) = 2.619 , *p* = .016) and 20 µg/µL (*t*(22) = 3.516, *p* = .002). Mice infused with raclopride at 20 µg/µL also trended towards a longer investigation time than saline infused animals during the third habituation session (*t*(22) = -1.985, *p* = .06). Unlike the estrous cycle analyses for the STFP (see Results; Table S3), the estrous cycle analysis for the ODT was likely underpowered to detect any effects, given the small sample sizes across phases and one empty cell (saline treated, estrus phase; n = 0; Table S6). We therefore so not draw conclusions about estrous effects on the ODT from this analysis. Interestingly, the initial reduction in investigation by DH raclopride did not affect the females’ olfactory discrimination of the CIN and COC diets. There were no effects of the estrous cycle for the NDI scores or total investigation data from habituation to test (or at separate phases), likely due to the small number of mice per phase, for each treatment group (Table S6).

**Figure S14.** DH raclopride olfactory discrimination task (ODT). Female mice received a single DH microinfusion of either saline vehicle (*n* = 12), or the dopamine (DA) D2-type receptor antagonist raclopride at 18 μg/μL (*n* = 11) or 20 μg/μL (*n* = 12). Infusions were administered 10 minutes prior to four habituation phases and one test phase. During the habituation phases (black bars) mice were exposed to two jars (covered with wire mesh to prevent consumption) containing the same food odors [i.e., two jars containing 1% ground cinnamon (CIN), or two jars containing 2% ground cocoa (COC)]. For the test session (gray bars) one of the formerly presented familiar diets was exchanged for a novel diet (i.e., mice were exposed to both CIN and COC). Each phase was five minutes in duration, and was administered consecutively in the home cage. For graph A: DH raclopride effects on female percent of novel diet investigation (NDI) scores ([time investigating novel diet / total time investigating both diets] x100); data are presented as mean **+** SEM; **p* < 0.05, ****p* < 0.001, averaged NDI scores of the four habituation phases versus test NDI scores. For graph B: DH raclopride effects on female total investigation [time investigating familiar diet + time investigating novel diet]; data are presented as mean ± SEM; **p* < 0.05, ***p* < 0.01, T = 0.05 < *p* < 0.1, in comparison to saline infused control animals.

**Table S6.** Sample sizes (*n*’s) for each estrous phase, per treatment group, for the olfactory discrimination task (ODT) DH raclopride experiments.

| **Olfactory discrimination task (ODT)** | | | |
| --- | --- | --- | --- |
|  | Proestrus | Estrus | Diestrus |
| Saline | *n* = 5 | *n* = 0 | *n* = 7 |
| Raclopride at 18 μg/μL | *n* = 5 | *n* = 1 | *n* = 5 |
| Raclopride at 20 μg/μL | *n* = 3 | *n* = 3 | *n* = 6 |

**DISCUSSION**

**Effects of DH Raclopride on Behavior during the Social Interactions of the STFP Task.**

We found that total activity was reduced by DH raclopride in female but not male OBS mice, both sexes showing a paralleled increase in time spent engaging in solitary inactivity. Both sexes showed a reduction in total social behavior, whereby males and females both showed a reduction in social investigatory and agonistic behaviors. However, reductions in agonistic-type behaviors were more pronounced in males, whereas reductions in social investigatory-type behaviors were more pronounced in females. There was a reduction in the dominance score for both sexes.

Critically, oronasal investigations were unaffected by DH raclopride in either sex, which is not surprising given that this is a reciprocal behavior which the DEM animal initiates often. Importantly, the fact that oronasal investigation was unaffected by drug treatment implies that the social learning impairment observed in females that received 18 μg/μL or 20 μg/μL of raclopride in the DH could not be explained by a decreased exposure to the socially transferred diet odor.

DH raclopride reduced total social behavior for both males and females. However, there were sex-specific effects of drug treatment on agonistic and investigatory-related behaviors. In males, there was a decrease in total agonistic behaviors, whereby agonistic behaviors delivered by the OBS to the DEM were reduced, including dominant behavior and boxing. In parallel with this, there was an increase in agonistic behaviors received, including submissive behaviors. This resulted in a reduced dominance score for raclopride treated males. There was also an overall reduction in male non-agonistic social investigation, including body and anogenital investigation. Hence, while male agonistic behaviors and social investigatory behaviors were both reduced by DH raclopride, effects on agonistic behaviors were greater, likely because males display more agonistic-type behaviors to begin with (see results on sex differences in saline-infused mice).

Female OBS social behaviors were also affected by DH raclopride. Drug treatment reduced female total social investigation, including body and anogenital investigation, as well as following the DEM. Interestingly, DH raclopride affected certain female agonistic behaviors. Females are generally less aggressive than males [37-40], and instead of engaging in male-typical overt aggressive behaviors (e.g., attacks, boxing), females are more likely to use a more non-attack agonistic approach, such as aggressive grooming and pushing down [2, 41-43]. In this study, female agonistic behavior delivered was reduced, including dominant behavior, which resulted in a reduced dominance score. Hence, together with social learning in the STFP, DH raclopride also reduced many female social behaviors, including social investigatory behaviors, as well as specific agonistic behaviors.

DA D2-type receptors have been implicated in many social behaviors such as social play [44], maternal behavior [45, 46], aggressive behavior [47, 48] and partner preference formation [49, 50]. Unfortunately, few studies have investigated the specific brain regions involved in the DA D2-type receptor mediation of social behaviors. One study found that DA D2 receptor levels increase in the mouse hippocampus following a social defeat [51]. Studies conducted on other mesocorticolimbic brain regions have found that non-aggressive mice show lower DA D2 receptor expression throughout the nucleus accumbens (NAc), as compared to aggressive mice [52]. Additionally, there is increased DA D2 receptor density in the NAc and dorsolateral caudate putamen of wild subordinate male rats [53]. This study adds to the literature above, where now hippocampal DA D2-type receptors have been shown to mediate social behaviors during home cage social interactions between same-sex familiar mice in the STFP.

The dominance scores of both males and females infused with raclopride were negative values, which imply that in both sexes the treated OBS were the subordinate animals of the pair during the social interactions. These DH results are consistent with the systemic results of Choleris et al., 2011 [1] which found that Raclopride injections resulted in a reduced dominance score at all doses [1]. These results are also in line with the DH results of Matta et al., 2017 [5], which found that males and females infused with the D1-type antagonist SCH23390 were more subordinate towards the DEM. Our results on social hierarchies are also consistent with a study which found that administering a DA D2-type receptor antagonist to socially dominant mice (but not subordinate animals) attenuates social dominance, and leads to more stable social hierarchies [54]. Hence, we show here that hippocampal DA D2-type receptors are also involved in social dominance-type behaviors.

It could be tempting to speculate that the reduction in dominance by DH raclopride lead to the impairment in social learning in the STFP, however, the fact that all doses of raclopride similarly reduced dominance in both sexes whereas only two doses blocked social learning and only in females, makes this explanation unlikely. Previous studies found no correlation between dominance score and the strength of a socially acquired food preference in female mice even though analysis of covariance indicated that increased subordination may be linked to an enhanced (not reduced) duration of the expression of a socially learned food preference [2]. Thus, while it seems unlikely that the effects of DH raclopride on social learning found here are directly driven by a reduction in the dominance score, further research on the role of dominance in the social transmission of information is warranted.

This study found that blocking hippocampal DA D2-type receptors resulted in a reduction in total activity for female, but not male mice. This overall decrease in female total activity was paralleled by an increase in time spent engaging in non-social non-locomotor behaviors, including solitary inactivity. Similarly, male non-social non-locomotor behaviors (i.e., solitary inactivity) were increased, and in addition, male self-grooming was reduced. DH raclopride also led to an increase in non-social behaviors in both sexes. Given the role of DA in regulating locomotion [55], such reductions in activity have been consistently reported after administration of DA receptor antagonists, including hippocampally [1, 5]. Since female total activity levels were similar across all Raclopride doses, and because Raclopride reduced total activity at all doses, even the doses that did not impair social learning, it is unlikely that the reduction in total activity could directly account for the social learning impairment found in this study. Additionally, we did not observe any abnormal stereotypic behaviors, which can be found at higher doses of drug treatment [e.g. 6]. Furthermore, oronasal investigation was unaffected by any dose, for either sex, which suggests that the social learning impairment of the female OBS could not be explained by a reduced exposure to the socially transferred diet odor. Hence, performing a full ethological analysis allowed us to determine that dorsal hippocampal DA D2-type receptors only influenced specific social and non-social behaviors, which contributed to the decrease in total activity levels.

The sex differences in various social behaviors found in this study suggest that DA in the hippocampus sex-specifically regulates social interactions through D2-type receptors. These results are in accordance with our previous hippocampal DA work, which found sex differences in social behaviors in response to DA D1-type receptor antagonism [5]. The exact mechanisms of action underlying how gonadal hormones may be interacting with DA receptors to mediate sex-specific effects on social behaviors are unclear. Further studies examining the interactions of gonadal hormones such as estrogens, progesterone and testosterone with the hippocampal DA system in the mediation of social behavior are therefore needed.

An olfaction control study revealed that female mice infused with the two doses of raclopride that impaired social learning could still discriminate between the two flavored diets used in the STFP. Such findings are in line with the systemic study of Choleris et al., 2011 [1], and the DH results of Matta et al., 2017 [5], which found that mice treated with the highest dose of the D1-type antagonist SCH23390 that blocked social learning, could still discriminate between CIN and COC diets.

In conclusion, hippocampal DA D2-type receptors sex-specifically mediate social learning and social interactions, but not food intake in the STFP. Blocking hippocampal DA D2-type receptors affected both social and non-social behaviors in males and females. While there were effects of drug treatment on social investigatory and agonistic behaviors in both sexes, males showed a greater reduction in agonistic-type behaviors, whereas females showed a greater reduction in social investigatory-type behaviors. This study warrants further research on the possible hormonal interplay with the hippocampal DA system in the regulation of social behaviors.

**Supplementary References**

1. Choleris E, Clipperton-Allen AE, Gray DG, Diaz-Gonzalez S, Welsman RG. Differential effects of dopamine receptor D1-type and D2-type antagonists and phase of the estrous cycle on social learning of food preferences, feeding, and social interactions in mice. Neuropsychopharmacol. 2011;36:1689-702.

2. Clipperton AE, Spinato JM, Chernets C, Pfaff DW, Choleris E. Differential effects of estrogen receptor alpha and beta specific agonists on social learning of food preferences in female mice. Neuropsychopharmacol. 2008;33:2362–75.

3. Sanchez-Andrade G, James BM, Kendrick KM. Neural encoding of olfactory recognition memory. J Reprod Dev. 2005;51:547–58.

4. Clipperton-Allen AE, Lee AW, Reyes A, Devidze N, Phan A, Pfaff DW, Choleris, E. Oxytocin, vasopressin and estrogen gene expression in relation to social recognition in female mice. Physiol Behav. 2011;105:915-24.

5. Matta R, Tiessen AN, Choleris E. The role of dorsal hippocampal dopamine D1-type receptors in social learning, social interactions and food intake in male and female mice. Neuropsychopharmacol. 2017;42:2344-53.

6. Phan A, Suschkov S, Molinaro L, Reynolds K., Lymer JM, Bailey CDC, et al. Rapid increases in immature synapses parallel estrogen-induced hippocampal learning enhancements. Proc Natl Acad Sci USA. 2015;112:16018–23.

7. Valsecchi P, Galef BG Jr. Social influences on the food preferences of house mice (Mus musculus). Int J Comparat Psychol. 1989;2:245-56.

8. Chepkova A, Sergeeva O, Haas H. Long-lasting enhancement of corticostriatal transmission by taurine: role of dopamine and acetylcholine. Cell Mol Neurobiol..2005;25:767-76.

9. Etter G, Krezel W. Dopamine D2 receptor controls hilar mossy cells excitability. Hippocampus. 2014;24:725-32.

10. Hamada M, Higashi H, Nairn A, Greengard P, Nishi A. Differential regulation of dopamine D1 and D2 signaling by nicotine in neostriatal neurons. J Neurochem. 2004;90:1094-1103.

11. Yabuuchi K, Kuroiwa M, Shuto T, Sotogaku N, Snyder GL, Higashi H, et al. Role of adenosine A1 receptors in the modulation of dopamine D1 and adenosine A2A receptor signaling in the neostriatum. Neurosci. 2006;141:19–25.

12. Gunaydin LA, Grosenick L, Finkelstein JC, Kauvar IV, Fenno LE, Adhikari A, et al. Natural neural projection dynamics underlying social behavior. Cell. 2014;157:1535–51.

13. Jung E, Lee H, Sim H, Baik J. Cocaine-induced behavioral sensitization in mice: Effects of microinjection of dopamine D2 receptor antagonist into the nucleus accumbens. Experimental Neurobiol. 2013;22:224-31.

14. Krzyzosiak A, Szyszka-Niagolov M, Wietrzych M, Gobaille S, Muramatsu S, Krezel W. Retinoid X receptor gamma control of affective behaviors involves dopaminergic signaling in mice. Neuron. 2010;66:908-20.

15. Kurokawa K, Mizuno K, Ohkuma S. Possible involvement of type 1 inositol 1,4,5-trisphosphate receptors up-regulated by dopamine D1 and D2 receptors in mouse nucleus accumbens neurons in the development of methamphetamine-induced place preference. Neurosci. 2012;227:22-9.

16. Presti M, Gibney B, Lewis M. Effects of intrastriatal administration of selective dopaminergic ligands on spontaneous stereotypy in mice. Physiology & Behavior. 2004;80;433-9.

17. Young A, Dreumont S, Cunningham L. Role of nucleus accumbens dopamine receptor subtypes in the learning and expression of alcohol-seeking behavior. Neurobiol Learn Mem. 2014;108:28-37.

18. Blanchard RJ, Yudko EB, Rodgers RJ, Blanchard DC. Defense system psychopharmacology: an ethological approach to the pharmacology of fear and anxiety. Behav Brain Res. 1993;58:155–65.

19. Choleris E, Gustafsson JA, Korach KS, Muglia LJ, Pfaff DW, Ogawa S. An estrogen-dependent four-gene micronet regulating social recognition: A study with oxytocin and estrogen receptor-alpha and -beta knockout mice. Proc Natl Acad Sci USA. 2003;100:6192-7.

20. Oksjoki S, Sallinen S, Vuorio E, Anttila L. Cyclic expression of mRNA transcripts for connective tissue components in the mouse ovary. Mol Hum Reprod. 1999;5:803-8.

21. Byers SL, Wiles MV, Dunn SL, Taft RA. Mouse estrous cycle identification tool and images. PLoS ONE. 2012;7:e35538.

22. Caligioni CS. Assessing reproductive status/stages in mice. Current Protocols in Neuroscience. 2009;48:A.4I.1–A.4I.8.

23. Lymer J, Robinson A, Winters BD, Choleris E. Rapid effects of dorsal hippocampal G-protein coupled estrogen receptor on learning in female mice. Psychoneuroendocrinol. 2017;77:131–40.

24. Phan A, Suschkov S, Molinaro L, Reynolds K, Lymer JM, Bailey CDC, et al. Rapid increases in immature synapses parallel estrogen-induced hippocampal learning enhancements. Proc Natl Acad Sci USA. 2015;112:16018–23.

25. Paxinos G, Franklin KBJ. The mouse brain in stereotaxic coordinates. 2nd ed. Academic Press; 2001.

26. Choleris E, Clipperton-Allen A, Phan A, Valsecchi P, Kavaliers M. Estrogenic involvement in social learning, social recognition and pathogen avoidance. Front Neuroendocrinol. 2012;33:140–59.

27. Ervin KS, Mulvale E, Gallagher N, Roussel V, Choleris E. Activation of the G protein-coupled estrogen receptor, but not estrogen receptor α or β, rapidly enhances social learning. Psychoneuroendocrinol. 2015;58:51-66.

28. Moran MD. Arguments for rejecting the sequential Bonferroni in ecological studies. Oikos; 2003;100: 403–5.

29. Nakagawa S. A farewell to Bonferroni: the problems of low statistical power and publication bias. Behav Ecol. 2004;15:1044–45.

30. Rothman KJ. No adjustments are needed for multiple comparisons. Epidemiology; 1990;1:43–6.

31. Bales KL, Plotsky PM, Young LJ, Lim MM, Grotte N, Ferrer E, et al. Neonatal oxytocin manipulations have long-lasting, sexually dimorphic effects on vasopressin receptors. Neuroscience.2007;144:38–45.

32. Cushing BS, Razzoli M, Murphy AZ, Epperson PM., Le WW, Hoffman GE. Intraspecific variation in estrogen receptor alpha and the expression of male sociosexual behavior in two populations of prairie voles. Brain Res. 2005;1016:247–54.

33. Cushing BS, Wynne-Edwards KE. Estrogen receptor-α distribution in male rodents is associated with social organization. J Comp Neurol. 2006;494:595–605.

34. Litvin Y, Murakami G, Pfaff DW. Effects of chronic social defeat on behavioral and neural correlates of sociality: vasopressin, oxytocin and the vasopressinergic V1b receptor. Physiol Behav. 2011;103:393–403.

35. Murakami G, Richard H, Fontaine C, Ribeiro AC, Pfaff DW. Relationships among estrogen receptor, oxytocin and vasopressin gene expression and social interaction in male mice. Eur J Neurosci. 2011;34:469–77.

36. Ribeiro AC, Pfaff DW, Devidze N. Estradiol modulates behavioral arousal and induces changes in gene expression profiles in brain regions involved in the control of vigilance. Eur J Neurosci. 2009;29:795–801.

37. Anton AH. Effect of group size, sex and time on organ weights, catecholamines and behavior in mice. Physiol Behav. 1969;4:483–7.

38. Edwards DA. Post-neonatal androgenization and adult aggressive behavior in female mice. Physiol Behav. 1970;5:465–7.

39. Jacobson-Pick S, Audet MC, McQuaid RJ, Kalvapalle R, Anisman H. Social agonistic distress in male and female mice: changes of behavior and brain monoamine functioning in relation to acute and chronic challenges. PLoS ONE. 2013;8:e60133.

40. Miczek KA, Maxson SC, Fish EW, Faccidomo S. Aggressive behavioral phenotypes in mice. Behav Brain Res. 2001;125:167–81.

41. Clipperton-Allen AE, Almey A, Melichercik A, Allen CP, Choleris E. Effects of an estrogen receptor alpha agonist on agonistic behaviour in intact and gonadectomized male and female mice. Psychoneuroendocrinol. 2011;36:981–95.

42. Clipperton-Allen AE, Cragg CL, Wood AJ, Pfaff DW, Choleris E. Agonistic behavior in males and females: effects of an estrogen receptor beta agonist in gonadectomized and gonadally intact mice. Psychoneuroendocrinol.2010;35:1008–22.

43. Grant EC, Mackintosh JH. A comparison of the social postures of some common laboratory rodents. Behavior. 1963;21:246–59.

44. Wang R, Xu X, Zhu Q. Pubertal exposure to di-(2-ethylhexyl) phthalate influences social behavior and dopamine receptor D2 of adult female mice. Chemosphere. 2016 ;144 :1771–9.

45. Cai Y, Zhang X, Jiang T, Zhong H, Han X, Ma R, Wu R. 8-OH-DPAT enhances dopamine D2-induced maternal disruption in rats. J Comp Physiol A. 2022 ;17 :1-11.

46. Zhu X, Peng S, Zhang S, Zhang X. Stress-induced depressive behaviors are correlated with Par-4 and DRD2 expression in rat striatum. Behav Brain Res. 2011;223:329–35.

47. Mahadevia D, Saha R, Manganaro A, Chuhma N, Ziolkowski-Blake A, Morgan AA, et al. Dopamine promotes aggression in mice via ventral tegmental area to lateral septum projections. Nat Commun. 2021;12:1-12.

48. Aguilar M, Miñarro J, Pérez-Iranzo N, Simón VM. Behavioral profile of raclopride in agonistic encounters between male mice. Pharmacol Biochem Be. 1995;47:753-6.

49. Herschberger MR, Perkeybile AM. Effects of a D2 receptor antagonist on repeated pair bond formation in the male prairie vole. Horm Behav. 2022;141:105149.

50. Young KA, Liu Y, Gobrogge KL, Wang H, Wang, Z. Oxytocin reverses amphetamineinduced deficits in social bonding: evidence for an interaction with nucleus accumbens dopamine. J Neurosci. 2014;34:8499 –8506.

51. Montagud-Romero S, Reguilon MD, Roger-Sanchez C, Pascual M, Aguilar MA, Guerri C, et al. Role of dopamine neurotransmission in the long-term effects of repeated social defeat on the conditioned rewarding effects of cocaine. Prog Neuropsychopharmacol Biol Psychiatry. 2016;71: 144-54.

52. Couppis MH, Kennedy CH, Stanwood GD. Differences in aggressive behavior and in the mesocorticolimbic DA system between A/J and BALB/cJ mice. Synapse. 2008;62:715-24.

53. Lucas LR, Celen Z, Tamashiro KLK, Blanchard RJ, Blanchard DC, Markham C, et al. Repeated exposure to social stress has long-term effects on indirect markers of dopaminergic activity in brain regions associated with motivated behavior. Neurosci; 2004;124:449–57.

54. Yamaguchi Y, Lee YA, Kato A, Jas E, Goto Y. The Roles of dopamine D2 receptor in the social hierarchy of rodents and primates. Sci Rep. 2017;24:43348.

55. Boutrel B. A neuropeptide-centric view of psychostimulant addiction. Br J Pharmacol.2008;154:343–57.

56. Cooper SJ, Al-Naser HA. Dopaminergic control of food choice: contrasting effects of SKF 38393 and quinpirole on highpalatability food preference in the rat. Neuropharmacol. 2006;50:953–63.
